# A Novel Drastic Peptide Genetically Adapted to Biomimetic Scaffolds “Delivers” Osteogenic Signals to Human Mesenchymal Stem Cells

**DOI:** 10.3390/nano13071236

**Published:** 2023-03-30

**Authors:** Aglaia Mantsou, Eleni Papachristou, Panagiotis Keramidas, Paraskevas Lamprou, Alexandros Pavlidis, Rigini M. Papi, Katerina Dimitriou, Amalia Aggeli, Theodora Choli-Papadopoulou

**Affiliations:** 1 Laboratory of Biochemistry, School of Chemistry, Faculty of Sciences, Aristotle University of Thessaloniki, University Campus, 54124 Thessaloniki, Greece; mantsouav@chem.auth.gr (A.M.); epapachristou@chem.auth.gr (E.P.); pankerdim@chem.auth.gr (P.K.); pa.lamprou@yahoo.com (P.L.); pavlalex@chem.auth.gr (A.P.); rigini@chem.auth.gr (R.M.P.); 2 Laboratory of Chemical Engineering A’, School of Chemical Engineering, Faculty of Engineering, Aristotle University of Thessaloniki, University Campus, 54124 Thessaloniki, Greece; katdimdim@cheng.auth.gr (K.D.); aggeli@cheng.auth.gr (A.A.)

**Keywords:** biomaterial, scaffold, bone regeneration, BMP-2 peptide, osteogenesis, tissue engineering, elastin, silk fibroin, mussel-foot protein

## Abstract

This work describes the design, preparation, and deep investigation of “intelligent nanobiomaterials” that fulfill the safety rules and aim to serve as “signal deliverers” for osteogenesis, harboring a specific peptide that promotes and enhances osteogenesis at the end of their hydrogel fibers. The de novo synthesized protein fibers, besides their mechanical properties owed to their protein constituents from elastin, silk fibroin and mussel-foot adhesive protein-1 as well as to cell-attachment peptides from extracellular matrix glycoproteins, incorporate the Bone Morphogenetic Protein-2 (BMP2) peptide (AISMLYLDEN) that, according to our studies, serves as “signal deliverer” for osteogenesis. The osteogenetic capacity of the biomaterial has been evidenced by investigating the osteogenic marker genes *ALP*, *RUNX2*, *Osteocalcin*, *COL1A1*, *BMPR1A*, and *BMPR2*, which were increased drastically in cells cultured on scaffold-BMP2 for 21 days, even in the absence of osteogenesis medium. In addition, the induction of phosphorylation of intracellular Smad-1/5 and Erk-1/2 proteins clearly supported the osteogenetic capacity of the biomaterial.

## 1. Introduction

Bone tissue has an inherent ability to regenerate when it is subjected to small lesions, but large defects, such as ones resulting from injury or surgery, lack this regeneration capacity [1,2]. Common treatments for these bone losses include autogenic or allogenic bone grafts and metal implants, all of which have their limitations: autogenic grafts are often difficult to acquire due to insufficient donor sites, allogenic grafts carry the risk of immunological rejection, and metal implants often have issues of integration with the surrounding live tissue, while in all cases, additional surgery is required, thus increasing the pain and risk of infection at the site [3,4]. Therefore, recent research is focusing on the development of safe biomaterials which can serve as scaffolds for the attraction of stem cells and the regeneration of damaged tissue after injection or implantation at the site [5]. As a temporary structure for the induction of bone formation, a scaffold needs to mimic the mechanical and biochemical properties of bone tissue, have good porosity and pore interconnectivity for nutrient supply, be biocompatible and biodegradable, and provide the necessary signals for cell differentiation [5].

Biomaterials based on fibrous proteins have gained popularity in tissue engineering thanks to their biocompatibility, tunable structure and function, and biodegradability. Silk-elastin-like polypeptides (SELPs) are a group of genetically engineered block copolymers that consist of repeated units of a silk fibroin-like peptide (GAGAGS) and an elastin-like peptide (VPGXG, where X can be any amino acid except proline) [6]. The silk-like part mimics the sequence in *Bombyx mori* silk fibroin and tends to self-assemble into insoluble beta-sheets that provide mechanical strength and thermal and chemical stability [7]. In parallel, the elastin-like part mimics the human tropoelastin sequences, which undergo a reversible, temperature-dependent phase transition. Thus, above a certain temperature (T_t_), they shift from soluble disordered polypeptides to aggregates of beta-spirals, which are characterized by high elasticity [8]. Additionally, the properties of the SELPs can be finely tuned by varying the X residue (charge, hydrophobicity, and crosslinking sites), the silk/elastin ratio, and the total molecular weight of the polypeptides [7]. Both proteins have been proven to cause little immunogenicity, cytotoxicity, or inflammation [7,9,10], which makes them ideal for the construction of scaffolding materials. Silk fibroin scaffolds embedded with human mesenchymal stem/stromal cells (hMSCs) and growth factors such as Bone Morphogenetic Protein-2 (BMP-2) have been fabricated in a way that enhanced bone regeneration more effectively than available clinical treatments [11,12].

Another category of fibrous proteins with useful properties for scaffold fixation and cell attachment are “Mussel Adhesive Proteins (MAPs)”, or “*Mytilus edulis* foot proteins (Mefps)”. These proteins are the main components of the “byssus”, a structure that is composed of a bundle of filaments with an adhesive plaque at the end, with which the mussel adheres strongly to organic and inorganic surfaces in an aqueous environment [13,14]. This adhesion mechanism relies on the high content of 3,4-dihydroxyphenyl-L-alanine (DOPA) residues in these proteins [14]. Various cells, including epiphyseal cartilage, osteosarcoma cells [15], neuronal cells [16], and human breast cancer cells [17], have been shown to attach successfully to Mefp-coated surfaces, which additionally indicates that Mefps are not toxic to cells. Mefp-1 has been shown to promote attachment and proliferation of keratinocytes and chondrocytes as efficiently as the widely used adhesives poly-D-lysine, fibronectin, and collagen, while at higher concentrations, it has been shown to support the adhesion of soft and hard tissues (skin–skin, skin–cartilage, and skin–bone) [13]. Therefore, Mefps have the capacity to improve cell binding and proliferation in scaffolds while being non-toxic and biodegradable. In fact, PCL/PLGA scaffolds coated with Mefp or Mefp–RGD peptide fusion have been fabricated that induced the binding, proliferation, and osteogenic differentiation of human adipose-derived stem cells (hADSCs) and aided bone formation in rat calvaria [18].

In the research of cell differentiation, attention has been focused on short peptides, which are small-molecular-weight active molecules that can regulate gene expression and affect proliferation, differentiation, and apoptosis [19]. The direction of pluripotent cell differentiation has been found to be affected by the peptide structure and concentration [20]. In multiple studies, short peptides (3–10 aa long) have promoted the differentiation of stem or progenitor cells to neuronal, lung, pancreatic, immune cells, and osteocytes [21,22,23,24,25,26]. For example, the W9 peptide (YCWSQYLCY) has been found to induce the osteogenic differentiation of the MC3T3-E1 murine line of immature osteoblasts and human mesenchymal stem cells via the p38 MAPK, Erk1/2, and Smad-1/5/8 pathways [25,27]. In previously published research in our laboratory, it has been shown that a short peptide from the carboxyterminal region of growth factor BMP-2, AISMLYLDEN, induces the osteogenic differentiation of hMSCs and the mineralization more effectively than the full-length protein [28,29].

In this project, a novel biocompatible scaffold was synthesized that combined, for the first time, the sequence, structure, and properties of human elastin, silk fibroin, and mussel-foot proteins with an inherent capacity to induce osteogenesis. This scaffold consisted of protein fibers, which were synthesized de novo at a genetic level, to contain functional peptides for cell attachment from fibronectin and laminin A2, a heparin-binding peptide for integration in the ECM, and the drastic peptide AISMLYLDEN from BMP-2. This biomaterial supported the growth of human dental pulp stem/stromal cells (hDPSCs) and induced the osteogenic signaling pathways necessary for their differentiation into osteoblasts and the mineralization of the ECM within 21 days.

## 2. Materials and Methods

### 2.1. De Novo Synthesis of Genes with Tandem Peptide Repeats

The DNA building blocks were constructed via a variant of PCR that involves sets of semi-complementary primers [8]. All primer sequences are listed in Table S1. Deep Vent DNA polymerase (New England Biolabs, Ipswich, MA, USA) was used in all PCR reactions for high performance and accuracy. The composition and conditions of the reactions are listed in Tables S2 and S3. The PCR products were checked by electrophoresis in 2% *w*/*v* agarose gels, with 1X GelRed Nucleid acid stain (Biotium, Fremont, CA, USA) and FastGene 50 bp DNA marker (Nippon Genetics, Düren, Germany). Three identical PCR reactions were performed for the synthesis of each building block, then the products were combined and purified with QIAquick PCR purification kit (QIAGEN, Hilden, Germany).

Each building block was then cloned into pET29c expression vector. For this purpose, both the vector and the purified PCR products were digested with restriction endonucleases NdeI and XhoI (New England Biolabs, Ipswich, MA, USA). Each PCR product was subjected to brief heat shock (incubation at 70 °C for 7 min, then incubation on ice for 7 min), then 40 U NdeI and 40 U XhoI were added, and the reaction was incubated at 37 °C for 3 h. The product was purified with the QIAquick PCR purification kit (QIAGEN, Hilden, Germany). For the digestion of the vector, three identical reactions were carried out. Each reaction was prepared with the same protocol as described for the PCR products, but the reactions took place at 37 °C for 15 min. Then, the vector’s 5′ termini were dephosphorylated by addition of 5 U Antarctic Phosphatase (New England Biolabs, Ipswich, MA, USA) and incubation at 37 °C for 1 h. The linear, dephosphorylated vector was purified from 1% *w*/*v* agarose gel using the QIAquick Gel extraction kit (QIAGEN, Hilden, Germany). The vectors with each DNA building block were ligated using T4 DNA ligase (New England Biolabs, Ipswich, MA, USA). For this purpose, the PCR product and the vector were mixed at 7:1 volume ratio and incubated with 400 U T4 DNA ligase at 16 °C for 16–18 h. Competent TOP10 *E. coli* were transformed with the product of the ligation reaction, and multiple colonies were selected for screening by plasmid DNA isolation (Nucleospin Plasmid Mini kit, Macherey Nagel, Düren, Germany) and digestion with NdeI and XhoI. The plasmids of all positive colonies were subjected to DNA sequencing.

The assembly of the DNA building blocks sequentially, in order to form the complete genes, was performed with the PRe-RDL method (Recursive Directional Ligation by Plasmid Reconstruction) [8]. Firstly, both plasmids were digested with 10 U BglI (New England Biolabs, Ipswich, MA, USA), an endonuclease that has a single recognition site within pET29c, at 37 °C for 15 min. The linear plasmids were purified from 1% *w/v* agarose gel. Afterwards, one plasmid was digested with AcuI and the second with BseRI. The reactions were carried out using 5 U AcuI or 5 U BseRI (New England Biolabs, Ipswich, MA, USA) at 37 °C for 15 min. The AcuI-digested plasmid was further dephosphorylated with 5 U Antarctic Phosphatase (New England Biolabs, Ipswich, MA, USA) at 37 °C for 1 h. The desired fragments from each plasmid were purified from 1% *w/v* agarose gels, and then, the complementary ends produced were ligated using T4 DNA ligase (New England Biolabs, Ipswich, MA, USA) in the following manner: BglI end–BglI end and AcuI end–BseRI end. AcuI/BglI-digested plasmid and BseRI/BglI-digested plasmid were mixed at 1:1 volume ratio and incubated with 400 U T4 DNA ligase at 16 °C for 16–18 h. Competent TOP10 *E. coli* were transformed with the product of the ligation reaction, and multiple colonies were screened by digestion with NdeI and XhoI.

### 2.2. Overexpression and Purification of the Polypeptides

For the overproduction of the polypeptides, *E. coli* BL21 (BE3) bacterial cells were used as a heterologous expression system. *E. coli* BL21 was transformed with each plasmid, and colonies were inoculated in Luria Bertani medium with 50 μg/mL kanamycin and grown for 16–18 h (overnight) at 37 °C and 130 rpm. Large-scale cultures (4 L) were performed by inoculating overnight cultures into flasks with 500 mL medium at 1:500 subculturing ratio. The cultures were incubated at 37 °C and 130 rpm until OD_600_ = 0.5–0.6, then 1 mM ITPG was added to induce overexpression. Samples were collected before and 5 h after induction for sodium dodecyl sulfate-polyacrylamide gel electrophoresis (SDS-PAGE). After 5 h, the cells were collected by centrifugation at 4000 rpm for 30 min at 4 °C and washed with pH 7.5 solution containing 10 mM Tris and 50 Mm NaCl. The cells were resuspended in pH 7.5 lysis buffer (10 mM Tris, 50 Mm NaCl, and 7 M GndHCl) and lysed by sonication in the following conditions: 70–100 cycles of 5-s ultrasound pulses of 50% intensity followed by 20 s pauses on ice. The cell lysates were centrifuged at 4000 rpm for 30 min at 4 °C, then the supernatant was subjected to nickel affinity chromatography. Protino Ni-NTA agarose beads (Macherey-Nagel, Düren, Germany) were equilibrated with lysis buffer (10 mM Tris, 50 Mm NaCl, and 7 M GndHCl, pH 7.5) and incubated with the lysis supernatant at 4 °C with rotation for 16–18 h. Afterwards, washes with increasing concentrations of imidazole (Tris 10 mM, NaCl 50 mM, 7 M GndHCl, and 30–200 mM, pH 7.5), and elutions with pH 7.5 solution containing Tris 10 mM, NaCl 50 mM, 7 M GndHCl, and 250 mM imidazole were performed. Samples from all the purification steps were dialyzed against ddH_2_O to remove guanidine hydrochloride and were subjected to SDS-PAGE.

### 2.3. Crosslinking and Formation of Scaffolds

The purified polypeptides were crosslinked using hexamethylene diisocyanate (HDI), a bifunctional agent that links lysines by the formation of a covalent bond between each isocyanate group of HDI, and an ε-amino group of a lysine residue HDI is an aliphatic di-isocyanate, which is resistant to hydrolytic degradation and offers ultraviolet stability. In this work, HDI was used as described in Martin et al. (2009) [30], in DMSO/DMF at ratio of 80:20, which ensures the inhibition of isothiocyanates hydrolysis that would give rise to subsequent side reactions and probably also to aldehyde formation. Specifically, the lyophilized polypeptides were mixed at equal weights (according to Table 2) and suspended in DMSO/DMF 80%:20% *v*/*v* solution. HDI was added at “polypeptide/crosslinker” molar ratio of 1:0.665, and the reaction took place at room temperature for 3 h with rotation. Afterwards, the organic solvents were removed by dialysis against ddH_2_O, and the crosslinked polymers were lyophilized. Porous scaffolds were formed by salt-leaching [1]. For this purpose, the crosslinked scaffolds were mixed with NaHCO_3_ at weight ratio of 1:10, suspended in ddH_2_O, and incubated at 37 °C for 24–48 h. The salt was removed by mild washing with pre-warmed (37 °C) 1X PBS.

### 2.4. Evaluation of Surface Morphology by Scanning Electron Microscopy (SEM)

The lyophilized crosslinked polymers were suspended in ddH_2_O, spread on borosilicate glass coverslips (VWR International, Radnor, PA, USA), and subjected to salt-leaching to form porous scaffolds. Then, they were washed mildly with 1X PBS (37 °C) to remove the salt and lyophilized. Finally, the scaffolds were carbon coated and observed under SEM (JEOL J.S.M. 840A, Tokyo, Japan) at the Electron Microscopy and Structural Characterization of Materials Laboratory of the Department of Physics at Aristotle University of Thessaloniki.

### 2.5. Evaluation of Rheological Parameters

A stress-controlled AR-G2 rheometer by TA Instruments (New Castle, DE, USA) was employed, which was thermostated at ±0.1 °C. A wide range of rheological measurements was carried out, in each case with a fresh sample to avoid any pretreatment effect unless otherwise stated: dynamic oscillatory strain sweeps, dynamic oscillatory frequency sweeps, dynamic oscillatory time sweeps in the linear viscoelastic regime (LVR), from which values of elastic (G′) and viscous (G″) moduli are obtained, and steady-state flow steps from which values of dynamic viscosity values are a function of shear rate, are derived. Calibration samples (standard oils) were also run, which were found to have values within 5% of their expected values, thus proving good operation of the instrument.

The measurements were performed at the A’ Chemical Engineering Laboratory of the Department of Chemical Engineering at Aristotle University of Thessaloniki. For this purpose, 6 mg/mL and 2 mg/mL suspensions of each crosslinked scaffold in DMEM were prepared, as well as 6 mg/mL of each uncrosslinked scaffold. Strain sweeps were performed on the crosslinked biomaterials at 37 °C, with application of 0.01–100% strain at 1 Hz frequency, to determine the Linear Viscoelastic Region (LVR). Then, time sweeps were performed within the LVR (at 3% strain) on the “crosslinked” biomaterials at 37 °C and on the “uncrosslinked” biomaterials at 25 °C and at 37 °C for 150 s. Temperature sweeps were performed on the “crosslinked” biomaterials at the range of 10–40 °C. Finally, flow measurements (flowstep) were performed at both states of the materials (crosslinked and uncrosslinked) at 37 °C. Strain sweeps and time sweeps were performed at two independent time points (April 2022 and July 2022). All measurements were carried out with different aliquots of the same sample in order to avoid preshearing effects and also repeated with multiple new sample preparations to show reproducibility. In total, >20 separate ELP samples were studied in detail rheologically, and their consistent, typical behavior is presented here with representative data.

### 2.6. Culture of Human Dental Pulp Stem/Stromal Cells and Differentiation on the Scaffolds

Human Dental Pulp Stem/Stromal Cells (hDPSCs) are a valuable source of multipotent stem cells, and studies highlight their capacity to in vitro differentiate into active osteoblasts [31]. For the purposes of this study, human hDPSCs were kindly provided by Associate Professor A. Bakopoulou from the School of Dentistry, Aristotle University of Thessaloniki. The cells had been established from third molars of young healthy donors, aged 18–24, with the enzymatic dissociation method described in Bakopoulou et al. (2015) [32]. The samples had been collected in accordance with all the relevant guidelines and regulations and had been approved by the Institutional Review Board of the Aristotle University of Thessaloniki (Nr. 66/18 June 2018). All the donors signed an informed consent form.

The hMSCs (hDPSCs) used in this study were cultured in α-MEM supplemented with 15% *v*/*v* fetal bovine serum (FBS), 100 U/mL penicillin, 100 μg/mL streptomycin, and 100 mM L-ascorbic acid phosphate (full α-MEM) in a cell culture incubator (37 °C and 5% CO_2_). For the differentiation assay, the scaffolds were subjected to salt leaching in 6-well plates for the formation of pores, as described in Section 2.3 and Martin et al. (2009) [30]. Prior to incubation at 37 °C, the scaffolds were sterilized by exposure to UV irradiation for 1 min and then incubated at 37 °C for 24 h. Exposure to UV for 1 min combined with the use of sterile reagents was selected as an adequate sterilization method for the scaffolds after preliminary animal studies in which the scaffolds sterilized in these conditions achieved the osteogenesis effect without inducing inflammation. Moreover, it was necessary to avoid prolonged exposure to ultraviolet light, which is known to affect the protein structure and the mechanical properties of nanofibrous structures [33,34]. Salt was removed by mild washing with pre-warmed (37 °C) 1X PBS, and cell culture medium with protease inhibitor cocktail for cell culture (Millipore Sigma, Burlington, MA, USA) was added for 30 min at 37 °C and 5% CO_2_. After reaching 80% confluency, hDPSCs were harvested with 0.05% (*w*/*v*) trypsin in PBS containing 0.02% (*w*/*v*) Na_2_EDTA and centrifuged at 750 g for 3 min. The cell pellet was resuspended in full-α-MEM and seeded onto the scaffolds and in empty wells at a density of 2 × 10^5^ cells/well. The cells were incubated overnight, then the culture medium was changed to osteogenesis medium in the appropriate wells. Where needed, BMP-2 peptide was added exogenously at 50 ng/mL final concentration. The plates were incubated in a cell culture incubator for 21 days with medium renewal every 2–3 days.

All media and reagents for cell culture were purchased from ThermoFisher Scientific (Waltham, MA, USA).

### 2.7. Cell Viability Assay (MTT)

The 3-(4,5-dimethylthiazol-2-yl)-2,5-diphenyltetrazolium bromide (MTT) assay is a quantitative method for measuring the metabolic activity of eukaryotic cells, which enables the estimation of their viability [35]. For this assay, the scaffolds were placed in 96-well plates at 2 concentrations, 1 mg/mL and 5 mg/mL, sterilized at ultraviolet light for 1 min, and subjected to salt leaching. These concentrations were chosen after previous MTT assays had been performed on HDFa cells cultured on 0.5 mg/mL and 1 mg/mL of the scaffolds (results under publication). Based on the observation that cell viability on these concentrations was similar to that of control cells, 1 mg/mL and a higher concentration, 5 mg/mL, were investigated in hMSCs in this study. Then, they were washed with pre-warmed (37 °C) 1X PBS and incubated in full α-MEM with protease inhibitor cocktail for cell culture (Millipore Sigma, USA) for 30 min at 37 °C and 5% CO_2_. hDPSCs were seeded onto the scaffolds and in empty wells at a density of 10^4^ cells/well and incubated for 3, 7, or 14 days. At each time point, MMT labeling reagent (Sigma-Aldrich, USA) was added to each well at final concentration of 0.5 mg/mL in 1X PBS, and the plates were incubated at tissue culture conditions for 3 h. The insoluble formazan was dissolved with 100% DMSO for 45 min at 37 °C, and the absorbance was measured against blank (DMSO) at 570 nm and a reference filter at 630 nm in a microplate reader.

### 2.8. Alizarin Red Staining Assay

Calcium deposits in the extracellular matrix were stained with Alizarin Red S (Sigma-Aldrich) 21 days after culture of hDPSCs on scaffold with BMP-2 peptide and without peptides. For this assay, cells were seeded in 6-well plates on porous scaffolds, as described in Section 2.7, and cultured for 21 days at 37 °C and 5% CO_2_ with appropriate medium changes. On the 21st day, the medium was removed, and the cells were washed with 1X PBS and fixed in 2.5% glutaraldehyde solution (Millipore Sigma, Burlington, MA, USA) at room temperature for 30 min. Afterwards, they were washed with sterile distilled H_2_O, incubated in 2% *w*/*v* Alizarin Red S solution (pH 4.1–4.3) (Millipore-Sigma, Burlington, MA, USA) at 25 °C for 30 min, and washed again thoroughly with sterile distilled water to remove excess dye. Photographs of the stained tissue were taken at 10× *g* magnification with a Nikon DS-Fi3 microscope camera (Tokyo, Japan).

Quantification was performed by dissolving the bounded Alizarin Red with cetylpyridinium chloride-CPC (10% *w*/*v*) in 10 mM Na_2_HPO4 (pH = 7) for 2 h at 37 °C. Optical absorption was measured at 550 nm with a microplate reader (Biotek Plate Reader, Winooski, VT, USA).

### 2.9. Morphological Observation of hMSCs by Scanning Electron Microscopy-SEM

For SEM imaging of cells cultured on scaffolds, the scaffolds were placed on borosilicate glass coverslips, as described in Section 2.4, inside 24-well plates and subjected to salt-leaching and sterilization, as described in Section 2.6. Then, cells were seeded on the scaffolds at density of 2 × 10^4^ cells per well in full α-MEM and incubated at 37 °C and 5% CO_2_ for 21 days. Afterwards, cells were washed in PBS, fixed with 3% glutaraldehyde (Millipore Sigma, Burlington, MA, USA) in 0.1 M sodium cacodylate containing 0.1 M sucrose, at pH 7.4, dehydrated using a graded series of ethanol concentrations, and remained under air-drying in the hood for 20–30 min before finally being carbon coated and observed under SEM (JEOL J.S.M. 840A, Tokyo, Japan).

### 2.10. Total RNA Isolation and cDNA Synthesis

Total RNA was extracted from hPDSCsusing the NucleoSpin RNA kit (Macherey-Nagel, Düren, Germany), according to the instructions. cDNA was synthesized using the PrimeScript RT reagent kit (Takara, Kusatsu, Shiga, Japan). A measure of 0.5 μg total RNA was used in each reaction, and the concentration of the produced cDNA was determined spectrophotometrically at 260 nm.

### 2.11. Real-Time PCR

Relative quantification of gene expression against reference genes *GAPDH* and *RPLPO* was performed on a StepOne Real time PCR System (Thermo Fisher Scientific, Waltham, MA, USA), using KAPA SYBR FAST qPCR Kit Master Mix (2×) ABI PRISM (KAPA BIOSYSTEMS, Wilmington, MA, USA) according to manufacturer’s protocol. Primers for all genes were purchased from Eurofins Genomics (Germany), and their sequences are listed in Table S4. The annealing temperature that was used for all the primer pairs was 60 °C. The qPCR program that was selected for all the reactions consisted of the following stages: an initial denaturation stage at 95 °C for 20 s, followed by 40 cycles of amplification (denaturation at 95 °C for 3 s and annealing and extension at 60 °C for 20 s), and, finally, a melt curve stage for each PCR product. All reactions were performed in triplicates. Relative expression of different gene transcripts was calculated by the ΔΔCt method. The Ct value of any gene of interest was normalized to the Ct values of two housekeeping genes (*GAPDH* and *RPLPO*).

### 2.12. Western Blotting

For the protein assays, hDPSCs were seeded and cultured on scaffolds for 21 days as described in Section 2.7. Then, the cells were washed with 1X PBS and lysed with RIPA lysis buffer (NaCl 150 mM, Tris-HCl pH 7.5 50 mM, NP-40 0.5% *v*/*v*, and deoxycholic acid (Na), 0.5% *v*/*v*) containing protease inhibitor cocktail (Millipore Sigma, USA), and the lysate was centrifuged at 11,000 rpm for 10 min at 4 °C. Samples of the protein extracts were reduced with β-mercaptoethanol in Laemmli buffer, separated by electrophoresis in 12% *w*/*v* SDS-polyacrylamide gels (50 μg of total protein/well), and transferred onto nitrocellulose membranes by semi-dry electrotransfer. The membranes were blocked in 5% *w*/*v* non-fat skimmed milk in PBS for 45 min and then incubated in primary antibody solution (antibody dilution 1:1000) for 16 h at 4 °C. After 3 washes with PBS containing 0.04% *v*/*v* Tween-20 (PBST), the membranes were incubated in secondary antibody solution (antibody dilution 1:2000) for 90 min at room temperature. For detection, the membranes were washed 3 times with PBST and incubated in 1X Alkaline phosphatase buffer (100 mM Tris-HCl pH 9.5, 100 mM NaCl, and 5 mM MgCl_2_) with substrates BCIP and NBT (Millipore Sigma, Burlington, MA, USA) at room temperature. Primary monoclonal antibodies for Erk1/2 (MAPK 42/44), phospho-Erk1/2 (phospho-MAPK 42/44), Smad-1, phospho-Smad-1/5, and secondary antibody (goat anti-rabbit IgG, alkaline phosphatase conjugated) were purchased from Cell Signaling Technology (Danvers, MA, USA). Primary polyclonal antibody for GAPDH was purchased from Santa Cruz Biotechnology (Dallas, TX, USA). Band intensities on the blots were quantified using the ImageJ 1.53 software, and ratios of phospho-Smad-1/5 to Smad-1 and of phospho-Erk1/2 to Erk1/2 were depicted on bar charts using the GraphPad Prism 8.2.1 software.

### 2.13. Statistical Analysis

The reported values on the graphs were expressed as mean ± standard deviation (SD) of experiments in triplicates (real-time PCR) and hexaplicates (MTT). Statistically significant differences between each test sample and the control were calculated using student’s *t*-tests for unpaired samples. Differences were considered statistically significant at the level of *p* ≤ 0.05. Statistical analyses and graphs were made using GraphPad Prism 8.2.1 software.

## 3. Results

### 3.1. Synthesis of the Recombinant Polypeptides

**Scheme 1:**
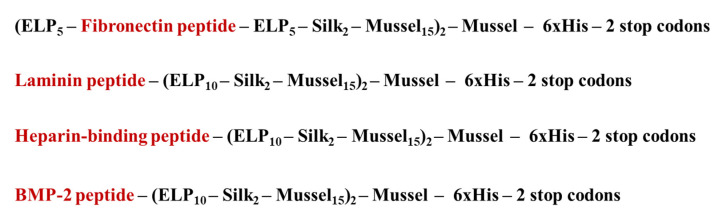
Depiction of the general structure of the synthesized genes.

The gene sequences for four recombinant polypeptides were synthesized, which combine building blocks derived from human tropoelastin [36], silk fibroin [36] and mussel-foot protein-1 [37] as well as cell-attachment peptides from human fibronectin [36] and laminin A2 [38], a heparin-binding peptide [39] to mimic the human extracellular matrix (ECM) and a short osteogenesis-inducing peptide from BMP-2, “AISMLYLDEN”. The building block composition of the genes (and the respective polypeptides) is depicted in Scheme 1, and the building block sequences are listed in Table 1. A coding sequence for a 6-histidine tag (6xHis tag) was incorporated at the 3′ end of each gene, followed by 2 stop codons. The RGD motif in the fibronectin peptide had been shown to be functional when incorporated within a polypeptide sequence [40,41]. However, this was the first time that scaffolds with the laminin peptide, a heparin-binding peptide, or a growth factor peptide were synthesized, thus those peptides were incorporated near the N′-terminus.

**Figure 1:**
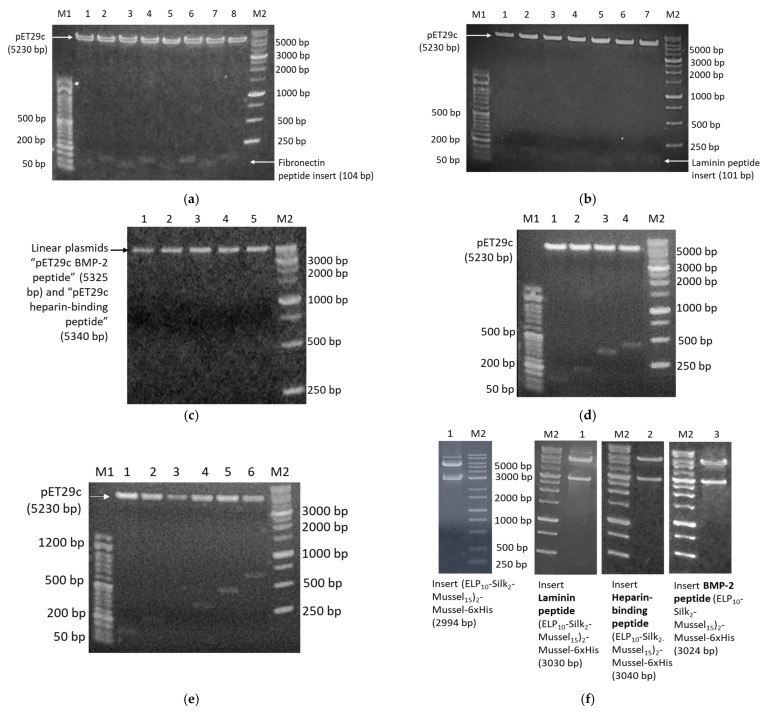

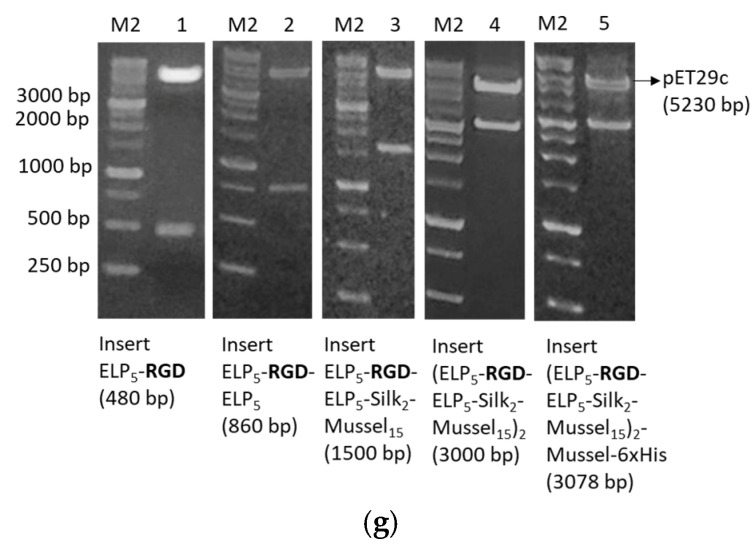
Step-by-step synthesis of recombinamer genes. (**a**–**c**) Electrophoresis in 1% *w*/*v* agarose gels of the recombinant plasmids containing building block sequences after digestion with NdeI and XhoI (**a**,**b**) or BseRI (**c**). In (**c**), lane 1 corresponds to digested pET29c-BMP2 peptide, and lanes 2–5 represent pET29c-heparin-binding peptide. (**d**–**f**) Step-by-step assembly of ELP_5_ and Silk_2_-Mussel_15_. Numbers in brackets state the length of the inserts. (**d**) Lane (1): pET29c-ELP_1_ insert (140 bp), lane (2): pET29c-ELP_2_ insert (215 bp), lane (3): pET29c-ELP_4_ insert (365 bp), and lane (4): pET29c-ELP_5_ insert (440 bp). (**e**) Lane (1): pET29c-Silk_1_ (164 bp), lane (2): pET29c-Silk_2_ (260 bp), lane (3): pET29c-Mussel_3_ (260 bp), lane (4): pET29c-Mussel_9_ (320 bp), lane (5): pET29c-Mussel_15_ (500 bp), and lane (6): pET29c–Silk_2_Mussel_15_ (710 bp). (**f**) pET29c–[(ELP_10_-Silk_2_-Mussel_15_)_2_-Mussel-6xHis] and final plasmids: pET29c–[laminin peptide-(ELP_10_-Silk_2_-Mussel_15_)_2_-Mussel-6xHis], pET29c–[heparin-binding peptide-(ELP_10_-Silk_2_-Mussel_15_)_2_-Mussel-6xHis], and pET29c–[BMP-2 peptide-(ELP_10_-Silk_2_-Mussel_15_)_2_-Mussel-6xHis] digested with NdeI/XhoI. (**g**) Step-by-step assembly of final plasmid pET29c–[ELP_5_-RGD-ELP_5_-Silk_2_-Mussel_15_)_2_-Mussel-6xHis]. Μ1: 50 bp DNA ladder and Μ2: 1 kb DNA ladder.

The DNA building blocks were synthesized via a PCR method that uses a pair of semi-complementary primers. Each primer contained part of the coding sequence for a DNA building block as well as restriction sites. The PCR products were inserted into the pET29c expression vector and propagated in TOP10 *E. coli* using molecular cloning techniques. The recombinant plasmids that were produced from the ligation reactions were screened by double digestion with NdeI and XhoI, and the products were evaluated by agarose gel electrophoresis. In cases where the insert was not visible on the gel, the plasmid was digested with BseRI, which has a single recognition and digestion site near the 3′ end of the insert (Figure 1a–e). After DNA sequencing of the inserts of the positive bacterial colonies, the plasmids containing the desired DNA sequences were selected for overexpression of the genes in BL21 *E. coli*. Colonies with verified DNA sequences are represented in lanes 1, 3, 5, and 7 of Figure 1a (pET29c-RGD), in lanes 1, 3, and 5 of Figure 1b (pET29c-laminin), and in all lanes of Figure 1c (pET29c-heparin-binding peptide and pET29c-BMP2 peptide). The assembly of the DNA building blocks sequentially to form the complete genes was performed with the PRe-RDL method. The recombinant plasmids that were produced after the final ligation reaction were screened by double digestion with NdeI and XhoI (Figure 1d–g).

All final nucleotide sequences of the genes are listed in the Supplementary Materials.

**Figure 2:**
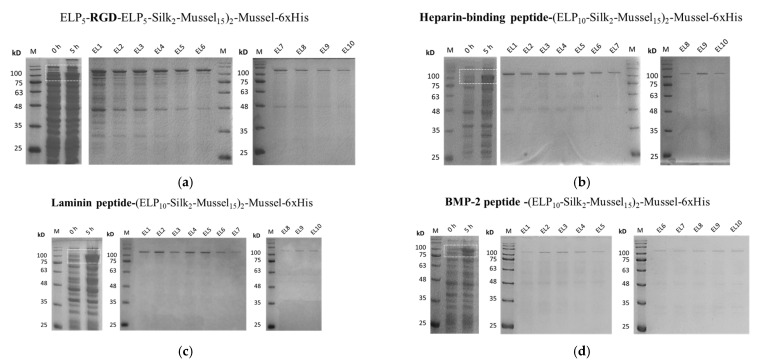
Electrophoresis in 10% *w*/*v* SDS-polyacrylamide gels of the fractions after overexpression (20 μL of cell extract per lane) and purification (10 μg of protein per lane) of the polypeptides from BL21 E. coli cells. (**a**) ELP_5_-RGD-ELP_5_-Silk_2_-Mussel_15_)_2_-Mussel-6xHis, (**b**) Heparin-binding peptide-(ELP_10_-Silk_2_-Mussel_15_)_2_-Mussel-6xHis, (**c**) Laminin peptide-(ELP_10_-Silk_2_-Mussel_15_)_2_-Mussel-6xHis, and (**d**) BMP-2 peptide -(ELP_10_-Silk_2_-Mussel_15_)_2_-Mussel-6xHis. 0 h: before induction of overexpression, 5 h: 5 h after induction of overexpression with 1 mM IPTG, and EL1-EL10: elutions with 250 mM imidazole. M: protein marker.

The polypeptides were overproduced and purified from BL21 (DE3) *E. coli* bacterial cells by Ni-NTA affinity chromatography. All final amino acid sequences are listed in the Supplementary Materials. The predicted molecular weight of the polypeptides was ~90,000 Da, and the corresponding bands after SDS-polyacrylamide gel electrophoresis were observed slightly above the 100-kD protein marker due to their fibrous nature (Figure 2).

**Figure 3:**
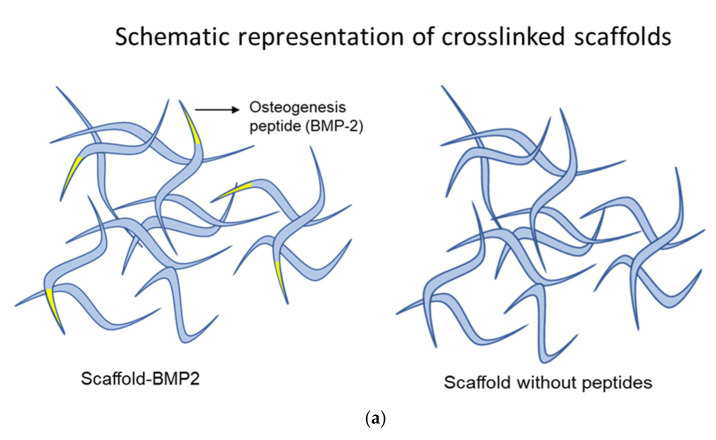

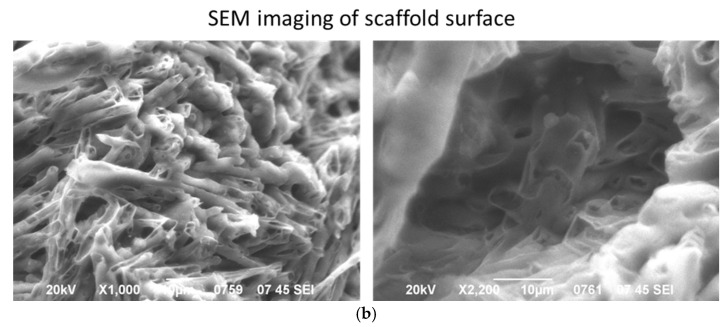
(**a**) Schematic representation of the three-dimensional scaffolds that were produced after the crosslinking reaction. (**b**) Imaging of the micromorphology of the porous surface of the scaffold without peptides by scanning electron microscopy (SEM) at 1000× *g* (left) and 2200× *g* (right) magnification. Each photograph has a scale bar of 10 μm.

For the formation of scaffolds, the polypeptides were crosslinked with the use of hexamethylene diisocyanate (HDI), which covalently links the ε-amino groups of lysine side chains. The polypeptides were mixed at equal weights and crosslinked to form two scaffolds: a scaffold with BMP-2 peptide (scaffold-BMP2) and a scaffold without BMP-2 peptide (scaffold without peptides), as described in Table 2. The crosslinked scaffolds were, then, subjected to salt leaching for the formation of pores. Figure 3a shows a schematic representation of the crosslinked scaffolds. The surface morphology of the scaffolds was assessed by scanning electron microscopy. Figure 3b shows a representative depiction of the surface micromorphology of the scaffold without peptides, as no differences in morphology and porosity were observed between the two scaffolds. The BMP-2 peptide, which is only 10 residues long, did not seem to influence the 3D structure of the scaffold or its rheological properties, as will be discussed in the following paragraph. The scanning revealed a porous surface (~50 μm pore size) with high interconnectivity.

### 3.2. Characterization of the Rheological Properties of the Scaffolds

**Figure 4:**
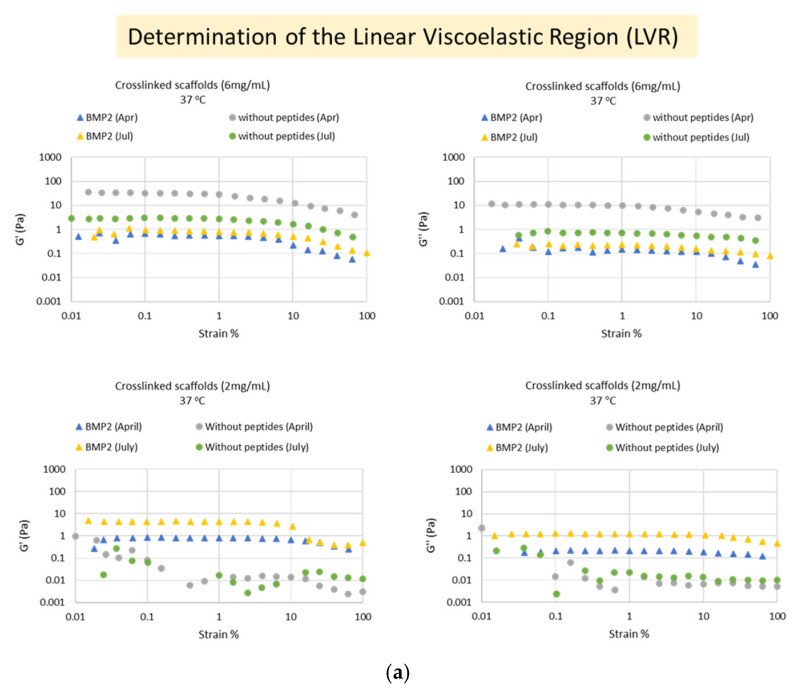

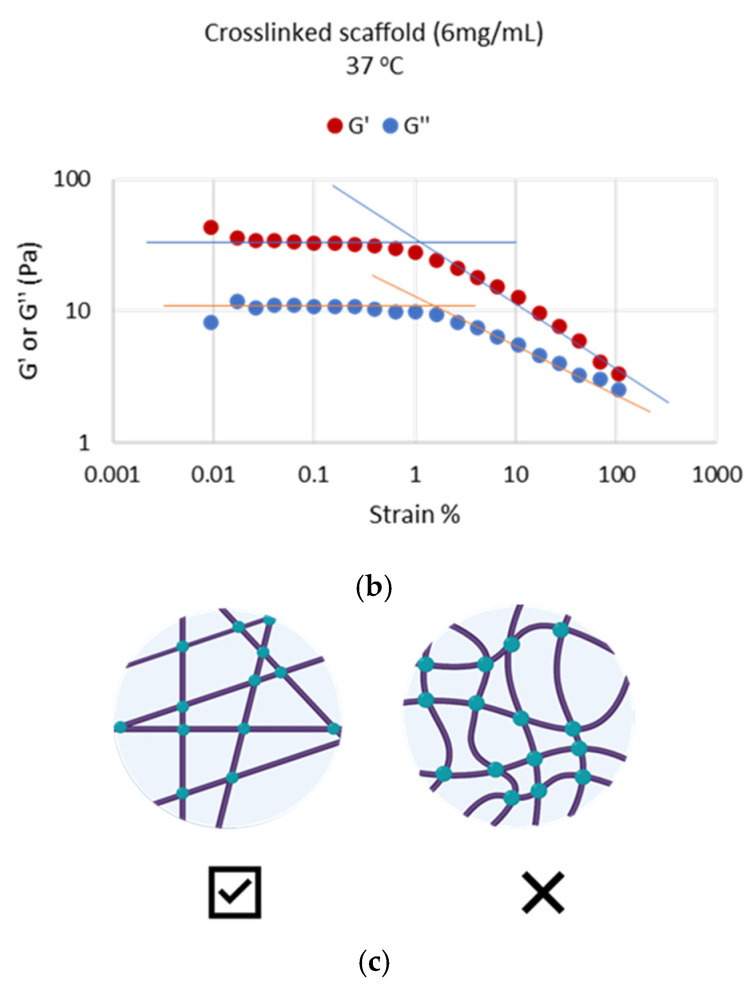
Determination of the Linear Viscoelastic Region of the crosslinked biomaterials. (**a**) Plots of elastic modulus (G′) or viscous modulus (G″) as a function of applied strain (%) in the crosslinked biomaterials (6 mg/mL in DMEM) at 37 °C. “BMP2”: scaffold-BMP2, “without peptides”: scaffold without peptides, “Apr”: April 2022 measurements, “Jul”: July 2022 measurements. (**b**) Plot of G′ and G″ as a function of applied strain (%) in the crosslinked biomaterials (6 mg/mL in DMEM) at 37 °C. The G′ to G″ ratio indicates the formation of elastic networks. (**c**) Schematic representation of the behavior of the biomaterials at 37 °C in DMEM. The “crosslinked” biomaterials have a small LVR region, indicating that they rather behave as semi-rigid (**a**), rather than fully flexible (**b**), polymers. Created with BioRender.com. All data correspond to different aliquots of the same samples in order to avoid preshearing effects or different sample preparations in order to show reproducibility.

The rheological behavior of the biomaterials before and after the crosslinking process—which will be referred to as “uncrosslinked” and “crosslinked”, respectively—were characterized at 25 °C and at 37 °C and 2 different concentrations, 2 mg/mL and 6 mg/mL, in the DMEM cell culture medium. Firstly, the Linear Viscoelastic Region of the crosslinked biomaterials (LVR) was determined to avoid the application of strain that might change the microstructure of the materials (strain outside the LVR) [42]. For this purpose, strain sweeps were performed at 0.01–100% strain (Figure 4a,b). It was observed that both biomaterials had a short LVR, which extended up to a maximum of ~15% strain, indicating that they behaved as semi-rigid polymer networks at the crosslinked state at 37 °C (Figure 4c). Additionally, both materials formed networks at the crosslinked state, as shown by their elasticity to viscosity ratio (G′ > G″) (Figure 4b).

**Figure 5:**
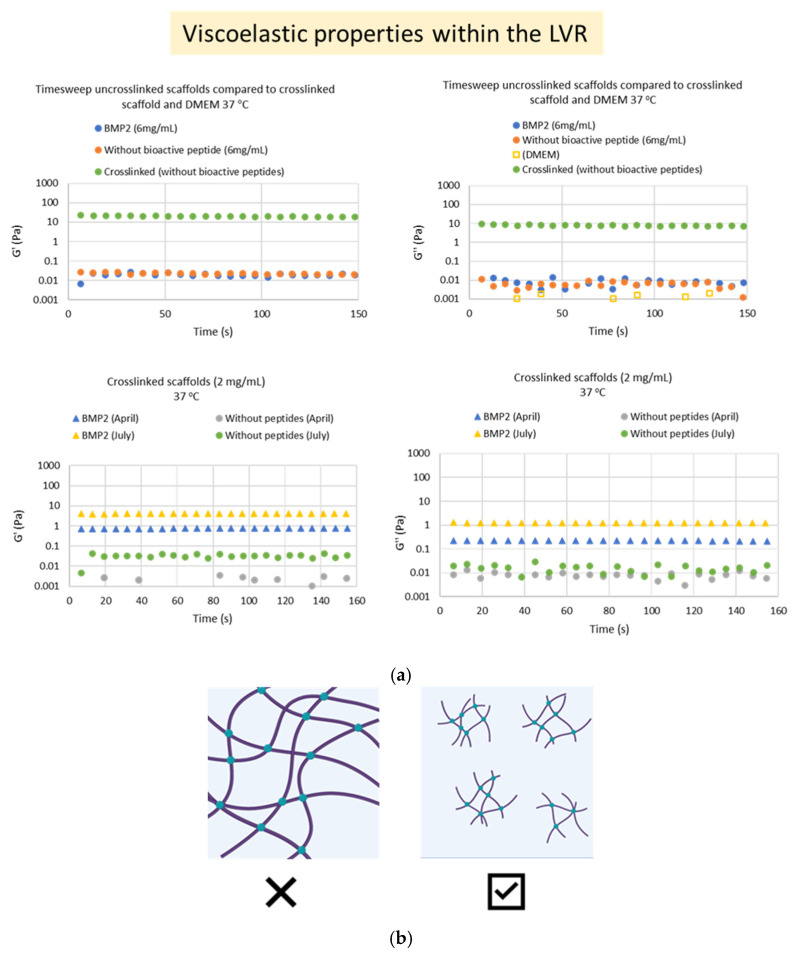
The viscoelastic properties of the crosslinked and uncrosslinked biomaterials, as measured within the LVR (at 3% strain). (**a**): Plots of elastic modulus (G′) and viscous modulus (G″) of the biomaterials, as a function of time (s) at 37 °C. “BMP2”: scaffold-BMP2, “without peptides”: scaffold without peptides, “Apr”: April 2022 measurements, “Jul”: July 2022 measurements. All data correspond to different aliquots of the same samples in order to avoid preshearing effects or different sample preparations in order to show reproducibility. (**b**): Schematic representation of the possible form of the scaffolds in DMEM at 37 °C, as islands of similar 3D networks instead of a continuous network. Created with BioRender.com.

The G′ and G″ moduli were determined with the application of strain within the LVR (3% strain) for 150 s (Figure 5a). The G′ and G″ values of the biomaterials in the crosslinked state were higher by 3 orders of magnitude compared to the uncrosslinked state (~10 Pa vs. ~0.01 Pa). The viscous modulus (G″) of the uncrosslinked biomaterials was slightly higher than the DMEM (0.01 Pa and 0.001 Pa, respectively). Therefore, after the crosslinking, they formed extensive 3-dimensional networks in DMEM at 37 °C exhibiting typical viscoelastic behavior (in most cases G′ = 3.5 ± 1.0 × G″).

It was interesting that different values were determined for the same material at two time points (April 2022 and July 2022). However, each measurement was conducted multiple times and given fully reproducible results. This led to the conclusion that the networks formed in DMEM at 37 °C were most likely not continuous networks; therefore, they did not span the whole volume of DMEM in the sample tube. Instead, they seemed to form individual islands of similar networks disconnected from each other in a sea of solvents. This model can explain the differences that were observed between the time points whilst, at the same time, the ratio G′/G″ remained similar (Figure 5b).

**Figure 6:**
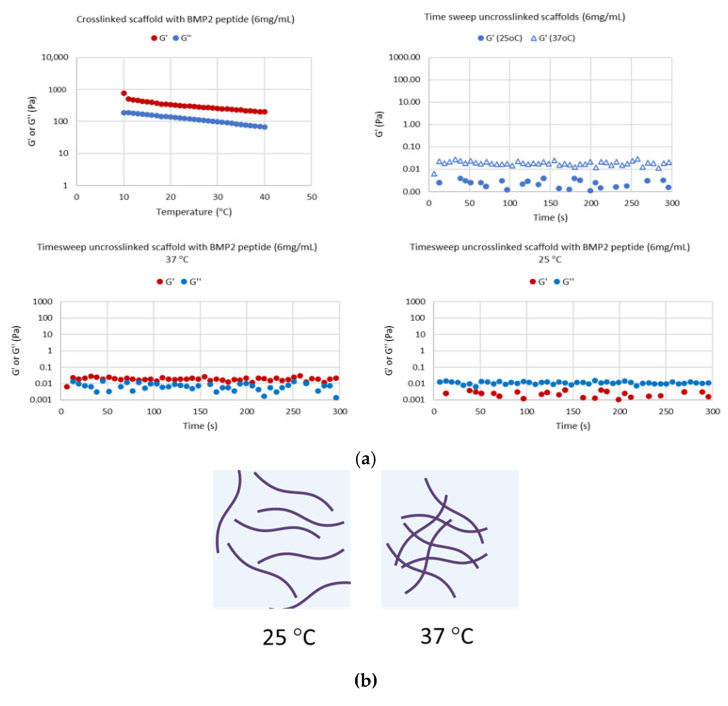
The effect of temperature on the viscoelastic properties of scaffold-BMP2, in the crosslinked and uncrosslinked state, at a concentration of 6 mg/mL in DMEM. (**a**) Temperature sweeps on the crosslinked scaffold-BMP2 at the range 10–40 °C and time sweeps on the uncrosslinked scaffold-BMP2 at 25 °C and 37 °C. The viscoelastic properties of crosslinked scaffolds showed a tendency to decrease with increasing temperature. In the uncrosslinked scaffolds, a shift in the G′/G″ ratio was observed between 25 °C and 37 °C, which indicated the possible formation of loose networks. All data correspond to different aliquots of the same samples in order to avoid preshearing effects or different sample preparations in order to show reproducibility. (**b**) Schematic representation of the potential transition observed in the “uncrosslinked” biomaterials between 25 °C and 37 °C (loose network formation). The schematic representations were created with BioRender.com.

Furthermore, to determine the effect of temperature on the behavior of the materials, the G′ and G″ were measured at the range 10–40 °C (Figure 6a). For crosslinked scaffolds, by increasing the temperature, the samples exhibited a typical temperature dependence for polymer networks, i.e., by increasing the temperature, the magnitudes of viscoelastic parameters decreased gradually at a rate of 10 Pa/°C. This contrasts with the typical phase-transition behavior expected of elastin-based polymers and is probably due to the crosslinking process and/or the presence of silk fibroin and mussel-foot protein peptide repeats in the fibers. However, the uncrosslinked scaffolds showed only the presence of viscous character and absence of network formation at 25 °C, whilst they did form a loose network when the temperature was raised to 37 °C, with a concomitant shift in the G′/G″ ratio between 25 °C and 37 °C (Figure 6b); it seems that the uncrosslinked ELPs demonstrate the peculiar temperature-induced phase transition expected of elastin-based biomaterials.

**Figure 7:**
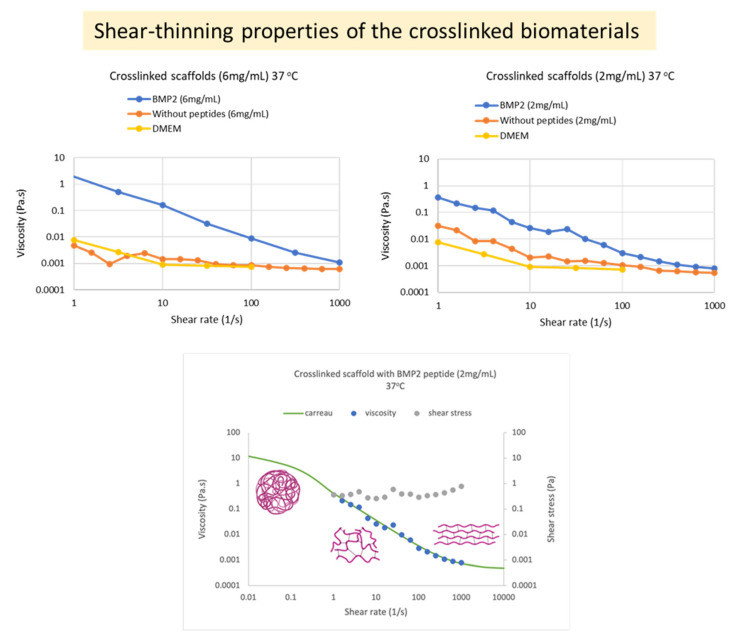
Determination of the shear-thinning properties of the crosslinked biomaterials at 37 °C (flowstep plots). The viscosity of the crosslinked scaffold-BMP2 decreases with increasing shear rate, indicating that the material is strongly shear thinning. The schematics show the proposed gradual acquisition of a common orientation of the fibers of the material with the increasing shear rate. Schematics were created with BioRender.com.

Finally, flow measurements showed that the crosslinked materials exhibited decreasing viscosity with increasing shear rate (Figure 7), thus they were strongly shear thinning (non-Newtonian fluids). In polymers, this phenomenon usually indicates the gradual acquisition of a common orientation of the fibers of the material with the application of increasing shear rate. Schematic representations of the proposed orientation of the fibers in the material gain with increasing shear rate are shown in Figure 7.

### 3.3. Assessment of Cytotoxicity of the Scaffolds on Human Dental Pulp Stem Cells

**Figure 8:**
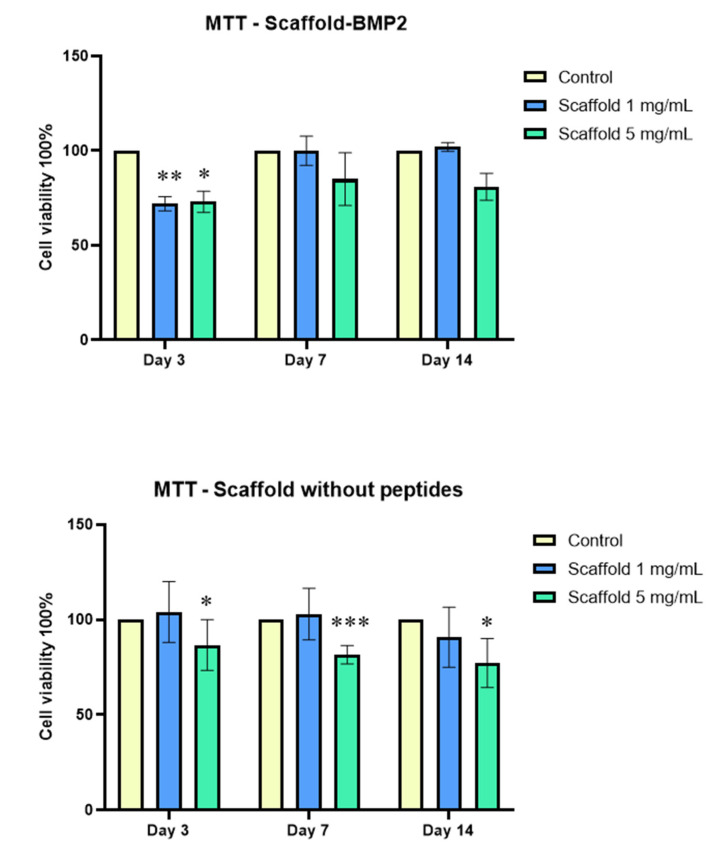
Evaluation of the viability of human dental pulp stem/stromal cells (hDPSCs) on different concentrations of scaffold-BMP-2 or scaffold without peptides by MTT assay. The assay was performed at 3 time points (3, 7, and 14 days of culture). The optical density was measured at 570 nm with a reference filter at 630 nm. “Control”: hDPSCs in full α-MEM (α-MEM supplemented with 15% *v*/*v* fetal bovine serum, 100 units/mL penicillin, 100 μg/mL streptomycin, and 100 mM L-ascorbic acid). The data are presented as mean± SD values of % cell viability. Asterisks (*), (**), and (***) indicate statistically significant differences (*p* ≤ 0.05, *p* ≤ 0.01, and *p* ≤ 0.001, respectively) compared to control cells.

The viability of human dental pulp stem/stromal cells (hDPSCs) on the scaffolds was assessed by MTT cytotoxicity assay (Figure 8). hDPSCs were cultured on scaffold-BMP2 or scaffold without peptides, which had been applied on the surface of the wells at 2 concentrations, 1 mg/mL or 5 mg/mL, for up to 14 days. MTT assay was performed at 3, 7, and 14 days. Both scaffolds were found to be non-toxic to hDPSCs. Specifically, after 7 and 14 days, the viability of cells on 1 mg/mL scaffold-BMP2 was equal to that of control cells, while at 5 mg/mL, a small reduction was observed (15–19%), which was not statistically significant (*p* < 0.05). Cell viability on a 1 mg/mL scaffold without peptides was almost equal to that of control cells, while a reduction was observed at 5 mg/mL after 14 days (*p* < 0.05). This may indicate that cells had begun to differentiate, thus decreasing cell number, and measured metabolic activity. The concentration of 1 mg/mL was chosen for further investigation of the capacity of the scaffolds to induce the osteogenic differentiation of hDPSCs.

### 3.4. The Scaffold That Contains the BMP-2 Peptide “AISMLYLDEN” Induces the Differentiation of hDPSCs into Osteoblasts and the Mineralization of the ECM Effectively

Following the results of the cytotoxicity assays, hDPSCs were cultured on 1 mg/mL scaffold-BMP2 or scaffold without peptides for 21 days, and then, the differentiation was evaluated by quantification of the expression of osteogenic gene markers by the investigation of osteogenic signaling (Smad-1/5/8 and Erk-1/2 pathways) and by staining of extracellular calcium deposits (Alizarin Red staining). For this experiment, 7 samples were used: (1) control cells (hDPSCs cultured in full α-MEM), (2) hDPSCs cultured in full α-MEM and treated with 50 ng/mL BMP-2 peptide, (3) hDPSCs cultured in osteogenesis medium, (4) hDPSCs cultured on the scaffold without peptides in full α-MEM, (5) hDPSCs cultured on the scaffold without peptides in osteogenesis medium, (6) hDPSCs cultured on scaffold-BMP2 in full α-MEM, and (7) hDPSCs cultured on scaffold-BMP2 in osteogenesis medium.

**Figure 9:**
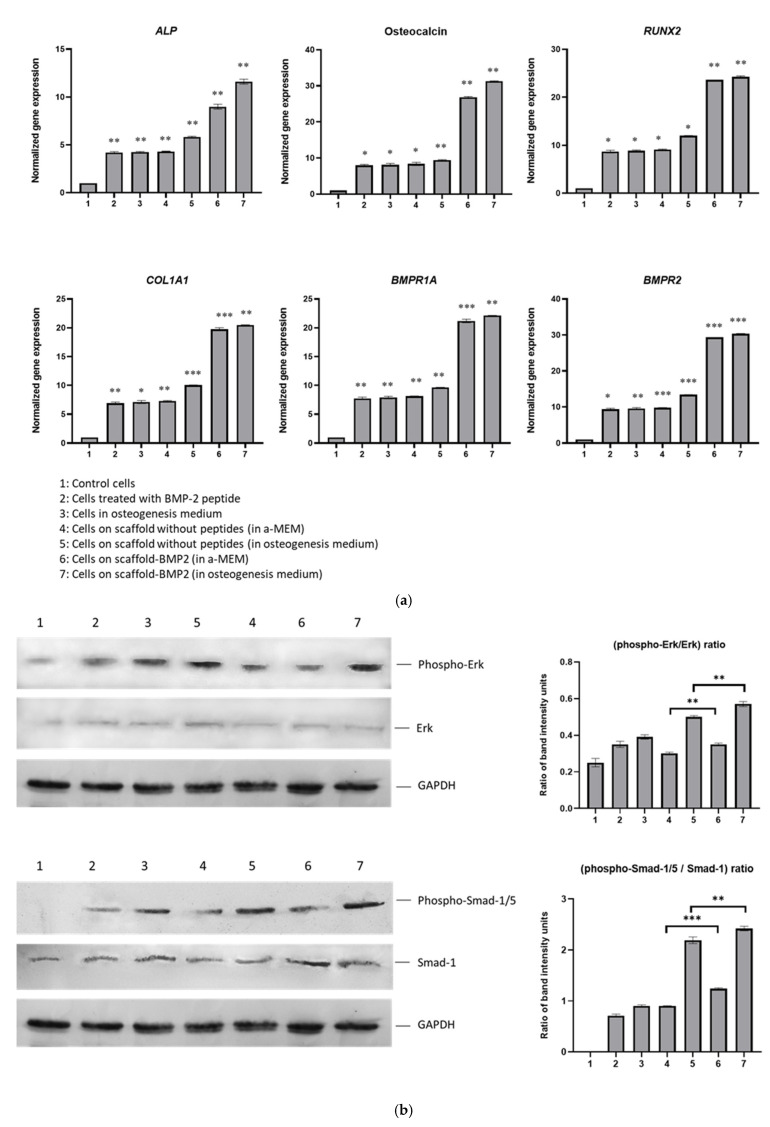
(**a**) Relative quantification of the mRNA levels of osteogenic markers *ALP*, Osteocalcin, *RUNX2*, *COL1A1*, *BMPR1A*, and *BMPR2* after 21 days of differentiation of hDPSCs on the scaffolds and without scaffold. The normalization of Ct values was performed against two housekeeping genes, *GAPDH* and *RPLPO*. (**b**) Western blotting against phospho-Erk1/2, Erk1/2, phospho-Smad-1/5, and Smad-1 in protein extracts after 21 days of differentiation of hDPSCs. The bar charts depict phospho-Erk/Erk and phospho-Smad-1/5/Smad-1 ratios after quantification of band intensities in the blots, using the ImageJ 1.53t software. The data are presented as the mean ± SD values (*n* = 3). Asterisks (*), (**), and (***) indicate statistically significant differences (*p* ≤ 0.05, *p* ≤ 0.01, and *p* ≤ 0.001, respectively) compared to control cells.

After 21 days of culture, total RNA and total protein were isolated from the cells for analysis. As shown in Figure 9a, all osteogenic marker genes had the highest expression in cells that had been cultured on scaffold-BMP2. In more detail, hDPSCs that had been treated with exogenous BMP-2 peptide, and hDPSCs that were cultured in an osteogenesis medium had 4-9 times higher expression of the osteogenic markers compared to control hDPSCs. In the cells that had been cultured on a scaffold without peptides in α-MEM medium, *ALP* expression was approximately 4.5 times higher, while the expression of the rest of the genes was 7–9 times higher than in control hDPSCs. The expression of these genes in cells that were cultured on this scaffold in the presence of osteogenesis medium was slightly higher than was observed in the presence of α-MEM. On the other hand, in cells that had been cultured on scaffold-BMP2, *ALP* expression was 9–11 times higher than in control cells, *RUNX2* expression was 24 times higher, *Osteocalcin* 27–31 times, *COL1A1* 20 times, *BMPR1A* 21–22 times, and *BMPR2* 29–30 times higher than in control hDPSCs. Thus, it was evident that scaffold-BMP2 at a concentration of 1 mg/mL was highly efficient in inducing the expression of genes involved in osteogenesis, even in the absence of an osteogenesis medium.

Intracellular levels of phospho-Erk1/2, Erk1/2, phospho-Smad-1/5, and Smad-1 were detected by Western blotting (Figure 9b). The phosphorylated forms of Erk1/2 and Smad-1/5 were higher when the cells were cultured on the scaffolds than without the scaffold, even in the absence of a differentiation medium. The phosphor-Erk to total Erk ratio was the highest in cells cultured on scaffold-BMP2 (46% higher in scaffold-BMP2/α-MEM and 113% higher in the scaffold-BMP-2/osteogenesis medium compared to control cells). Low phospho-Erk levels were detected in control cells. Similarly, the phospho-Smad-1/5 to Smad-1 ratio was the highest in cells cultured on scaffold-BMP2 (40% higher in scaffold-BMP2/α-MEM and 168% higher in the scaffold-BMP-2/osteogenesis medium compared to cells with BMP-2 peptide or cells in osteogenesis medium). Phospho-Smad-1/5 was undetected in control cells. These results verify that scaffold-BMP2 leads to efficient induction of both the Smad-1/5/8 and the Erk1/2 (Smad-independent) pathways of osteogenic signaling.

**Figure 10:**
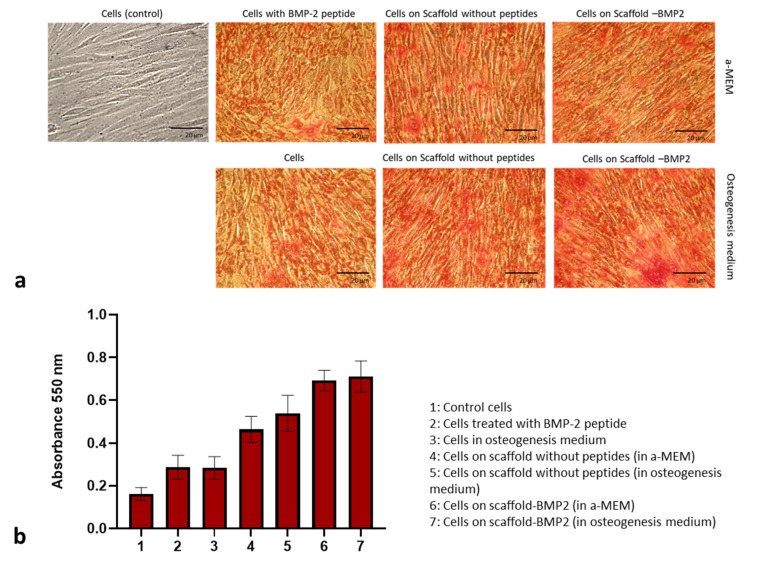
(**a**) Detection of calcium deposits in the extracellular matrix by Alizarin Red staining after 21 days of culture of hDPSCs on scaffolds and without scaffolds. The photographs were taken at 10× *g* magnification with a Nikon DS-Fi3 microscope camera, and 20 μm-scale bars have been included. (**b**) Quantification of Alizarin Red staining at 21 days. Optical absorption was measured at 550 nm with a microplate reader (Biotek Plate Reader).

In parallel, calcium deposits in the ECM increased markedly, as shown in Figure 10. Stained areas were abundant in hDPSCs cultured on the scaffold without peptides, but the most abundant in hDPSCs cultured on scaffold-BMP2. Interestingly, although the combination of scaffold-BMP2 and osteogenesis medium resulted in the most effective mineralization, scaffold-BMP2 alone was also able to induce efficient mineralization.

**Figure 11:**
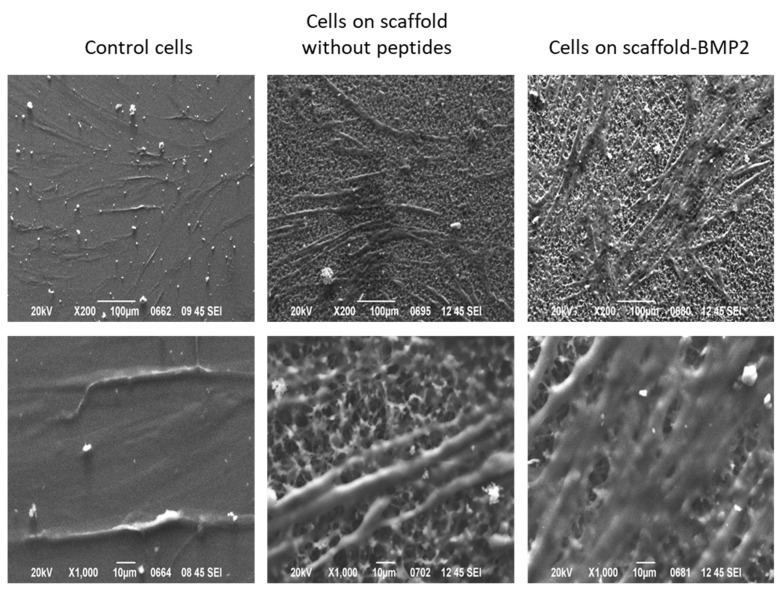
SEM microphotographs of control hDPSCs, hDPSCs cultured on scaffold without peptides and hDPSCs cultured on scaffold-BMP2 for 21 days. Photographs were taken at 200× *g* and 1000× *g* magnification.

Furthermore, hDPSCs were seeded on scaffold-BMP2 and scaffold without peptides, which had been placed on borosilicate glass coverslips, and they were cultured for 21 days. Then, the cells were observed with scanning electron microscopy (Figure 11). It seems that both scaffolds provide a favorable environment for the cells.

## 4. Discussion

Bone tissue engineering for the treatment of critical bone defects requires biomaterials with growth factor activity, so they have an enhanced ability to induce the differentiation of stem cells [43,44]. To address this issue, we attempted, for the first time, the design and synthesis of a novel biomaterial that contained functional peptides for cell attachment and inherent growth factor activity for osteogenic signaling. The core of the material was a combination of tandem peptide repeats from human tropoelastin (VPGVG/VPGKG), *Bombyx mori* silk fibroin [V(GAGAGS)_5_G] and *Mytilus edulis* mussel-foot protein-1 (AKPSYPPTYK), which have been thoroughly investigated as sources for scaffolding materials, thanks to their mechanical properties, biodegradability, and low immunological response [11,12,18,36,45,46,47,48]. The incorporation of integrin-binding peptides derived from ECM glycoproteins, such as “YAVTGRGDSPASSG” from human fibronectin, has been shown to improve cell attachment and proliferation on biomaterials [34,44]. Moreover, short peptides that can bind to cell surface integrins have been identified in laminins of the basal lamina [38,49]. Of these, the 12-residue peptide “YHYVTITLDLQQ” from laminin A2 has shown efficient attachment to human cells (fibroblasts and myoblasts), which is not inhibited by the presence of heparin and interacts with integrin-α2β1 expressed in human mesenchymal stem cells [50]. To enhance the capacity of our material to attract and bind mesenchymal stem cells, these fibronectin and laminin a2 peptide sequences were incorporated in separate fibers, which were then crosslinked to form a network (scaffold). Similarly, other fibers of the scaffold were designed to contain a synthetic peptide with the capacity to bind to heparin, “YPTQRARYQWVRCNP” [39], thus enabling the interaction of the scaffold with ECM proteoglycans and enhancing integration in the tissue. Finally, certain fibers displayed the drastic BMP-2 peptide “AISMLYLDEN” at their N′-terminal end (scaffold-BMP2) [28,29]. For comparison, a second scaffold was constructed, which did not contain fibers with the BMP-2 peptide (scaffold without peptides).

The evaluation of their rheological properties showed that both scaffolds at the crosslinked state formed extensive 3-dimensional networks at 37 °C, which exhibited typical viscoelastic behavior (G′ = 3.5 ± 1.0 × G″) and were stable with time (Figure 4 and Figure 5). These were most likely semi-rigid, individual islands of networks rather than a continuous network. The crosslinked scaffolds showed temperature dependence which was typical of most polymers, with G′ and G″ decreasing gradually with increasing temperature. However, there were also indications of loose network formation at the uncrosslinked state, as shown by a shift in G′/G″ ratio from 25 °C to 37 °C (Figure 6). The materials were strongly shear thinning at the crosslinked state (Figure 7), an important characteristic for injectable polymers. In nature, in vivo osteogenesis happens using mainly an intrinsic collagen 3D network as a scaffold. Previous systematic in vitro studies of collagen-based 3D matrices (hydrogels) have established that these matrices are characterized by values of rheological parameters (G′, G″ η) of the same order of magnitude and of the same range as the values we obtained with our scaffolds and present in this manuscript. Therefore, it is concluded that the networks produced in this work seem to provide appropriate natural-like biophysical (mechanical) cues to the surrounding cells during differentiation.

MTT assays showed that both crosslinked scaffolds, at a concentration of 1 mg/mL, exhibited excellent biocompatibility with human dental pulp stem/stromal cells (Figure 8). At 5 mg/mL of scaffolds, a slight reduction was observed compared to the control, which probably indicated the induction of osteogenic differentiation.

Bone tissue formation and regeneration are regulated primarily by Bone Morphogenetic Proteins (BMPs). One of its members, BMP-2, which has been extensively studied as an inducer of osteogenic differentiation, acts through binding to a dimeric Type II/Type I receptor (BMRP2/BMPR1A) to initiate various signaling cascades that eventually lead to the upregulation of transcription factors involved in osteoblast differentiation, such as *RUNX2*, *ID1*, and *OSX* [51]. These, in turn, are responsible for the production of enzymes and structural proteins that form the bone tissue extracellular matrix (e.g., alkaline phosphatase, collagen type I, osteocalcin, and osteopontin) [52]. These responses in the nucleus are the result of various signaling pathways, which have been categorized as canonical and non-canonical. In the canonical pathway of BMP-2, the activated receptors phosphorylate intracellular Smad-1/5/8 proteins, which then form a complex with co-Smad (Smad-4) that is transported to the nucleus and acts as a transcriptional regulator of osteogenesis-related genes [53]. Non-canonical pathways (Smad-independent) include the activation of MAP kinases, such as Erk, p38 MAPK, or Jnk, which translocate to the nucleus, where they activate ATF, c-Jun, or Fos, to regulate BMP target genes, such as *ALP* or *COL1A1* (collagen type I alpha 1 chain) [54].

To investigate the osteogenic capacity of the synthesized scaffolds, the expressions of genes *RUNX2*, *ALP*, *Osteocalcin*, *COL1A1*, *BMPR1A*, and *BMPR2* were quantified by real-time PCR in cells that had been cultured on them for 21 days. As mentioned above, *RUNX2* is one of the initial transcription factors activated by BMP-2 signaling, which is responsible for the further expression of other transcriptional regulators and effector proteins of osteogenesis. Such effectors are the enzyme alkaline phosphatase (encoded by the *ALP* gene) which hydrolyzes pyrophosphate and provides inorganic phosphate to promote mineralization [55], collagen type I chain a1 chain (*COL1A1*), the precursor for the formation of collagen type fibers [56] and osteocalcin, the most abundant non-collagenous protein in bone ECM, and, thanks to its high affinity to Ca^2+^, it has a critical role in mineralization [57]. For these reasons, these genes were selected in our study to be investigated as markers of osteoblast differentiation, along with the genes encoding the BMP2 receptors, type I (*BMPR1A*) and type II (*BMPR2*). Our results demonstrated that the BMP2-scaffold, even in the absence of osteogenesis medium, was the most efficient in inducing the upregulation of all marker genes, approximately 2–3 times more efficient than cells without scaffolds and cells on the scaffold without peptides (Figure 9a). More specifically, by comparing the expression of these genes between the cells cultured on the scaffold without peptides in α-MEM medium and the cells cultured on scaffold-BMP2 in α-MEM (samples “4” and “6”, respectively, in Figure 9a), we observed an, at least 2-fold, upregulation in the latter. Similarly, by comparing the cells on the same scaffolds but in osteogenesis medium (samples “5” and “7”), 2- to 3-fold upregulation of gene expression was seen in cells on scaffold-BMP2. The osteogenesis effect, in both scaffolds, was enhanced by the addition of the osteogenesis medium, as it is expected; however, the scaffold-BMP2 alone considerably induced the expression of these genes (in α-ΜΕΜ medium). These results indicate that the genetically incorporated sequence AISMLYLDEN was effectively displayed on the scaffold surface to bind to BMPRII/BMPRI receptors and activate signaling. Additionally, *BMPR1A* and *BMPR2* genes were upregulated, possibly resulting in a positive feedback loop of BMP-2 signaling.

Furthermore, phosphorylated and total Smad-1/5 and Erk-1/2 were detected by Western blotting in protein extracts on the 21st day of culture (Figure 9b). The calculation of phospho-Erk to Erk and phospho-Smad-1/5 to Smad-1 ratios showed that scaffold-BMP2 induced both pathways, but mostly the Smad-dependent pathway. The phospho-Smad-1/5 to Smad-1 ratio in cells cultured on scaffold-BMP2 in α-MEM was higher than that observed in scaffold without peptides (α-MEM) in cells in the osteogenesis medium and cells supplemented with exogenous BMP-2 peptide. In more detail, the phospho-Smad-1/5 to Smad ratio in cells cultured on the scaffold without peptides in α-MEM was almost equal to that of cells cultured in an osteogenesis medium without scaffolds, indicating that it had the capacity to induce the canonical osteogenesis signaling at a certain level. This effect was higher when the cells were cultured on scaffold-BMP2 in α-ΜΕΜ (with a statistically significant difference). In both scaffolds, the phosphorylation of Smad-1/5 was enhanced significantly by the addition of an osteogenesis medium due to the differentiation factors contained in it.

The phospho-Erk to Erk ratio was lower in cells cultured on a scaffold without peptides compared to cells cultured in an osteogenesis medium without scaffolds or cells treated with exogenous BMP-2 peptide. The ratio was increased when cells were cultured on scaffold BMP-2 in α-ΜΕΜ, and though it remained slightly lower than the level observed in cells in the osteogenesis medium, it was evidently increased compared to control cells. This shows that the Erk1/2 pathway was induced by the scaffold-BMP2, although probably to a lower extent than the Smad-1/5 pathway. In both scaffolds, the phosphorylation of Erk-1/2 was enhanced by the addition of the osteogenesis medium. In cells on scaffold-BMP2 in osteogenesis medium, the ratio was ~33% higher than the cells in the same medium without scaffolds, showing that the scaffold contributed to the induction of the pathway.

These results were accompanied by the staining of calcium cations in the extracellular matrix of hDPSCs cultured on scaffolds with Alizarin Red. The abundance of stained areas and the increased expression of alkaline phosphatase [58] verified that the scaffolds, especially scaffold-BMP2, resulted in the successful mineralization of the extracellular matrix.

**Scheme 2:**
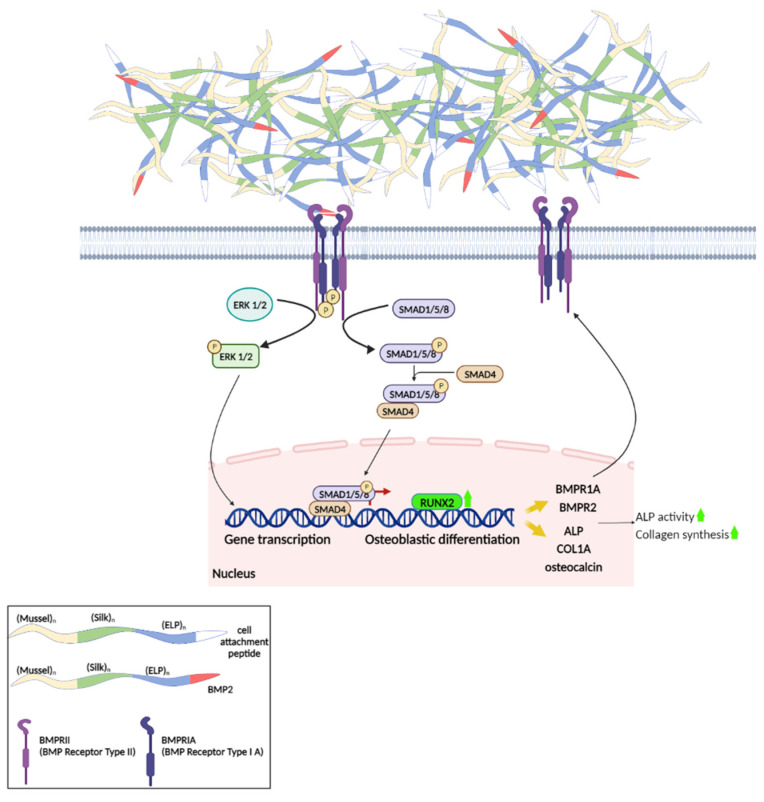
Schematic representation of the signal transduction induced by the scaffold-BMP2 in human dental pulp stem/stromal cells. Upon binding of the drastic BMP-2 peptide to the BMR1A/BMPR2 receptor complexes, the activated BMPR1A receptor (a serine–threonine kinase) phosphorylates and activates intracellular Smads-1/5/8 and Erk-1/2. Activated Smad-1/5/8 forms a complex with co-Smad (Smad-4), which translocates to the nucleus and acts as transcriptional activator of osteogenesis-inducing transcription factors. In the same way, the Erk-1/2 upon its phosphorylation after its receptor activation translocates similarly to the nucleus acting as a transcriptional activator of osteogenetic factors. In particular, both signaling pathways activate transcriptional factors such as *RUNX2*, which in turn enhance the production of enzymes and structural proteins involved in the formation of bone extracellular matrix (alkaline phosphatase -ALP-, collagen type I -Col1A-, osteocalcin, BMPR1A, and BMPR2). Schematic representation created with BioRender.com.

These findings strongly support that the scaffold-BMP2 induces the pathways of osteogenic signaling, leading to the upregulation of genes and the induction of osteogenic differentiation and mineralization (Scheme 2).

## 5. Conclusions

Nowadays, the development of biomaterials has entered dynamically the “nanotechnology era”. The current advances in nanobiomaterials research span a wide range of tissue engineering applications (both soft and hard tissues), drug delivery, disease detection, and disease treatment. However, the exertion of mechanical forces on the cells regarding cell–cell interaction as well as cell–substrate interaction, namely the mechano-biological aspects, emerges concerns about the safety of manufacturing and using nanobiomaterials, especially toxicological issues.

Within this work, we describe the design, synthesis, and extensive investigation of “intelligent nanobiomaterials” that fulfill the safety requirements and aim to serve as “signal deliverers” for osteogenesis by harboring a specific peptide that promotes and enhances osteogenesis at the end of their fibers. Our research project, taking into account the limited capacity of large bone lesions to regenerate, focused on the development of a biomimetic scaffold inspired by natural fibrous proteins with the potent inherent capacity to induce the osteogenic differentiation of stem cells and the formation of mineralized bone tissue.

Preliminary studies in animal models (mice) have implicated the injection of BMP-2 protein solution subcutaneously to achieve osteogenesis in vivo (unpublished results) based on Urist et al. (1965) [59]. BMP-2 is a dimer stabilized by a disulfide bridge and the maintenance of its conformation, which is vital to its functionality, is often difficult to secure during its handling and injection. This limitation is overcome by the use of the short BMP-2 peptide (genetically incorporated in our scaffold), which has long-term stability and was shown, in our experiments, to be effective in inducing osteogenesis. Currently running preliminary studies in mice have shown successful ectopic bone formation after injection of the scaffold-BMP2 without any sign of inflammation at the site (unpublished results and under further investigation). These results support the future use of the scaffold-BMP2 as an injectable biomaterial for the regeneration of bone from a patient’s autologous stem cells and, thus, the permanent treatment of bone tissue disruptions, such as fractures.

## Figures and Tables

**Table 1:** Nucleotide and amino acid sequences of building blocks.

Building Block	Nucleotide Sequence (5′ to 3′)	Amino Acid Sequence
ELP	GTGCCGGGCGTTGGTGTTCCGGGCGTCGGTGTCCCGGGTAAAGGCGTTCCGGGTGTTGGTGTTCCGGGTGTTGGC	VPGVGVPGVGVPGKGVPGVGVPGVG
Silk	GTGGGCGCGGGTGCCGGCAGCGGTGCAGGCGCTGGTTCTGGCGCTGGCGCGGGTTCCGGTGCCGGTGCAGGCTCCGGCGCCGGCGCTGGTTCTGGC	V(GAGAGS)_5_G
Mussel	GCGAAACCGAGCTATCCGCCGACCTATAAA	AKPSYPPTYK
Fibronectin (RGD) peptide	TATGCGGTGACCGGTCGTGGTGATAGCCCGGCCTCTAGCGGC	YAVTGRGDSPASSG
Laminin peptide	TATCATTACGTGACCATTACGCTGGATTTACAGCAA	YHYVTITLDLQQ
Heparin-binding peptide	TATCCGACCCAGCGTGCGCGCTATCAATGGGTGCGTTGCAACCCG	YPTQRARYQWVRCNP
BMP-2 peptide	GCGATTAGCATGCTGTATTTAGATGAAAAC	AISMLYLDEN
6xHis tag	CATCACCATCACCATCAC	HHHHHH

**Table 2:** Composition of crosslinked scaffolds.

Polypeptides	Scaffold without Peptides	Scaffold-BMP2
(ELP_5_-RGD peptide-ELP_5_-Silk_2_-Mussel_15_)_2_-Mussel-6xHis	+	+
Laminin peptide-(ELP_10_-Silk_2_-Mussel_15_)_2_-Mussel-6xHis	+	+
Heparin-binding peptide-(ELP_10_-Silk_2_-Mussel_15_)_2_-Mussel-6xHis	+	+
BMP-2 peptide-(ELP_10_-Silk_2_-Mussel_15_)_2_-Mussel-6xHis	-	+

## Data Availability

The data presented in this study are available on request from the corresponding author.

## Supplementary Materials

The following supporting information can be downloaded at: https://www.mdpi.com/article/10.3390/nano13071236/s1, Table S1: Primer sequences for the synthesis of the DNA building blocks by PCR; Table S2: Composition of PCR-based-ligation reactions; Table S3: Conditions of PCR-based-ligation reactions; Table S4: Primer sequences for real-time PCR; DNA and amino acid sequences.
