# Supplementary material for: A Novel Drastic Peptide Genetically Adapted to Biomimetic Scaffolds “Delivers” Osteogenic Signals to Human Mesenchymal Stem Cells
Source: Nanomaterials (Basel). 2023 Mar 30;13(7):1236. doi: 10.3390/nano13071236 (PMC10096854; doi:10.3390/nano13071236)
Supplement: Supplementary file 1 [file nanomaterials-13-01236-s001.zip › nanomaterials-2291300-supplementary.pdf]

Supplementary material

A Novel Drastic Peptide Genetically Adapted to Biomimetic Scaffolds "Delivers" Osteogenic Signals to Human Mesenchymal Stem Cells

Aglaia Mantsou [1], Eleni Papachristou [1], Panagiotis Keramidas [1], Paraskevas Lamprou [1], Alexandros Pavlidis [1], Rigini M. Papi [1], Katerina Dimitriou [2], Amalia Aggeli [2] and Theodora Choli-Papadopoulou [1,*]

[1] Laboratory of Biochemistry, School of Chemistry, Faculty of Sciences, Aristotle University of Thessaloniki, University Campus, 54124 Thessaloniki, Greece; mantsouav@chem.auth.gr (A.M.); epapachristou@chem.auth.gr (E.P.); pankerdim@chem.auth.gr (P.K.); pa.lamprou@yahoo.com (P.L.); pavlalex@chem.auth.gr (A.P.); rigini@chem.auth.gr (R.M.P.)
[2] Laboratory of Chemical Engineering A', School of Chemical Engineering, Faculty of Engineering, Aristotle University of Thessaloniki, University Campus, 54124 Thessaloniki, Greece; katdimdim@cheng.auth.gr (K.D.); aggeli@cheng.auth.gr (A.A.)
* Correspondence: tcholi@chem.auth.gr

Tables

Table S1. Primer sequences for synthesis of the DNA building blocks by PCR.

**Primer name**	**Sequence (5'-3')**
ELP forward	GACTGTCATATGGTTAGCCTGTTGCCGGTGAGGAGCGGTGTGGGCGTG CCGGGCGTTGGTGTTCCGGGCGTCGGTGTCCCGGGTAAAGGC
ELP reverse	AACCACCTCGAGCTGAAGAGCTGTGGTCTGGTGCCAACACCCGGAACA CCAACACCCGGAACGCCTTTACCCGGGAC
Silk forward	GACTGTCATATGGAGGAGCGGTGTGTGCGTGGGCGCGGGTGCCGGCAG CGGTGCAGGCGCTGGTTCTGGCGCTGGCGCGGGTTCC-
Silk reverse	AACCACCTCGAGCTGAAGAGCTGTGGTCTGGTGCCAGAACCAGCGCCG GCGCCGGAGCCTGCACCGGCACCGGAACCCGCGCCAGCGCC
Mussel forward	GACTGTCATATGGAGGAGCGGTGTGTGCGCGAAACCGAGCTATCCGCC GACCTATAAAGCCAAACCGTCTTATCCGCCGACGTATAAA
Mussel reverse	AACCACCTCGAGCTGAAGAGCTGTGGTCTGGTGCCTTTATACGTCGGC GGATAGCTCGGTTTTGCTTTATACGTCGGCGGATAAGACGGTTTGGC
Mussel-6xHis reverse	AACCACCTCGAGTTATTAGTGATGGTGATGGTGATGGCTGAAGCCACC AGAACCGCTGCCTTTATACGTCGGCGGATAAGACGGTTTGGC
Fibronectin (RGD) peptide forward	GACTGTCATATGGTTAGCCTGTTGCCGGTGAGGAGCGGTGTGGGCTAT GCGGTGACCGGTCGTGGTGAT
Fibronectin (RGD) peptide reverse	AACCACCTCGAGCTGAAGAGCTGTGGTCTGGTGCCGCTAGAGGCCGGG CTATCACCACGACCGGTCACCGCATA
Laminin peptide forward	GACTGTCATATGGAAAGCCTGTTGCCGGTGAGGAGCGGTGTGGGCTAT CATTACGTGACCATTACGCTGGATTTACAGCAA
Laminin peptide reverse	AACCACCTCGAGCTGAAGAGCTGTGGTCTGGTGCCTTGCTGTAAATCC AGCGTAATGGTCACGTAATGATA
Heparin-binding peptide forward	GACTGTCATATGGAAAGCCTGTTGCCGGTGAGGAGCGGTGTGGGCTAT CCGACCCAGCGTGCGCGCTATCAATGGGTGCGTTGCAACCCG
Heparin-binding peptide-reverse	AACCACCTCGAGCTGAAGAGCTGTGGTCTGGTGCCCGGGTTGCAACGC ACCCATTGATAGCGCGCACGCTGGGTCGGATA

BMP-2 peptide forward	GACTGTCATATGGAAAGCCTGTTGCCGGTGAGGAGCGGTGTGGGCGCGATTAGCATGCTGTATTTAGATGAAAAC
BMP-2 peptide reverse	AACCACCTCGAGCTGAAGAGCTGTGGTCTGGTGCCGTTTTCATCTAAATACAGCATGCTAATCGC

Table S2. Composition of PCR-based-ligation reactions.

**Reagent**	**Final concentration**
Forward primer	500 nM
Reverse primer	500 nM
Thermopol Buffer	1X (contains 2 mM $MgSO_4$)
$MgSO_4$	10 mM
dNTP mix	3 mM each
Deep Vent DNA polymerase	6 U
Sterile ddH_2O	Up to 50 μL

Table S3. Conditions of PCR-based-ligation reactions.

<table>
<tr><th>Stage</th><th>Temperature (°C)</th><th>Duration (min)</th><th>Number of cycles</th></tr>
<tr><td>Initial denaturation</td><td>95</td><td>3</td><td>1</td></tr>
<tr><td>Denaturation</td><td>95</td><td>1</td><td rowspan="10">20</td></tr>
<tr><td>Annealing (primer pairs):</td><td></td><td></td></tr>
<tr><td rowspan="2">• ELP</td><td>65</td><td>1</td></tr>
<tr><td>56</td><td>1</td></tr>
<tr><td>• Silk</td><td>72</td><td>1</td></tr>
<tr><td>• Mussel</td><td>65</td><td>1</td></tr>
<tr><td rowspan="2">• Mussel-6xHis</td><td>65</td><td>1</td></tr>
<tr><td>60</td><td>1</td></tr>
<tr><td>• RGD peptide</td><td>65</td><td>1</td></tr>
</table>

	62	1	
• Laminin peptide	65	1	
	58	1	
• Heparin-binding peptide	72	1	
• BMP-2 peptide	65	1	
	53	1	
Extension	72	1	
Final extension	72	5	1
Hold	4	∞	

Table S4. Primer sequences for real-time PCR.

**Primer**	**Nucleotide sequence (5’ to 3’)**
GAPDH forward	GCACCGTCAAGGCTGAGAAC
GAPDH reverse	TGGTGAAGACGCCAGTGGA
RPLPO forward	AATGTGGGCTCCAAGCAGAT
RPLPO reverse	TGAGGCAGCAGTTTCTCCAG
ALP forward	ATGGGATGGGTGTCTCCACA
ALP reverse	CCACGAAGGGGAACTTGTC
RUNX2 forward	TCATGGCGGGTAACGATGAA
RUNX2 reverse	GTGAAACTCTTGCCTCGTCC
COL1A1 forward	CAGTGTGGCCCAGAAGAACT
COL1A1 reverse	CCGCCATACTCGAACTGGAAT
Osteocalcin forward	CGGTGCAGAGTCCAGCAAA
Osteocalcin reverse	GGTAGCGCCTGGGTCTCTTC
BMPR1A forward	TATGGATGGGCAAATGGCGT
BMPR1A reverse	TCTGCCGCTATGAAACCAAGT
BMPR2 forward	AACCTGCAATTTCCCATCGAGA
BMPR2 reverse	CTGATAGTGCCAACCTCGCT
SOX9 forward	GGCAAGCTCTGGAGACTTCTG
SOX9 reverse	CCCGTTCTTCACCGACTTCC
COL2A1 forward	AGAATCCATCTGAGAATATGC
COL2A1 reverse	CCTCTTACTGCTATACCTTTAC
ACAN forward	GGAAGGGAGGGGAACCATTG
ACAN reverse	TGATGGCTGTCCACTGACAC

TGFBR1A forward	AGCAGCAGACAATAAAGACAATGG
TGFBR1A reverse	TGTGAAGATGGGCAAGACCG
TGFBR2 forward	CTGTAATGCAGTGGGAGAAGTAAAA
TGFBR2 reverse	AATTTCTGGTCGCCCTCGAT

Sequences

[(ELP$_5$-RGD-ELP$_5$-Silk$_2$-Mussel$_{15}$)$_2$-Mussel-6xHis]

Nucleotide sequence (3012 bp):

ATGGTTAGCCTGTTGCCGGTGAGGAGCGGTGTGGGCGTGCCGGGCGTTGGTGTTCCGGGCGTCG
GTGTCCCGGGTAAAGGCGTTCCGGGTGTTGGTGTTCCGGGTGTTGGCGTGCCGGGCGTTGGTGTT
CCGGGCGTCGGTGTCCCGGGTAAAGGCGTTCCGGGTGTTGGTGTTCCGGGTGTTGGCGTGCCGG
GCGTTGGTGTTCCGGGCGTCGGTGTCCCGGGTAAAGGCGTTCCGGGTGTTGGTGTTCCGGGTGTT
GGCGTGCCGGGCGTTGGTGTTCCGGGCGTCGGTGTCCCGGGTAAAGGCGTTCCGGGTGTTGGTGT
TCCGGGTGTTGGCGTGCCGGGCGTTGGTGTTCCGGGCGTCGGTGTCCCGGGTAAAGGCGTTCCGG
GTGTTGGTGTTCCGGGTGTTGGCTATGCGGTGACCGGTCGTGGTGATAGCCCGGCCTCTAGCGGC
GTGCCGGGCGTTGGTGTTCCGGGCGTCGGTGTCCCGGGTAAAGGCGTTCCGGGTGTTGGTGTTCC
GGGTGTTGGCGTGCCGGGCGTTGGTGTTCCGGGCGTCGGTGTCCCGGGTAAAGGCGTTCCGGGT
GTTGGTGTTCCGGGTGTTGGCGTGCCGGGCGTTGGTGTTCCGGGCGTCGGTGTCCCGGGTAAAGG
CGTTCCGGGTGTTGGTGTTCCGGGTGTTGGCGTGCCGGGCGTTGGTGTTCCGGGCGTCGGTGTCC
CGGGTAAAGGCGTTCCGGGTGTTGGTGTTCCGGGTGTTGGCGTGCCGGGCGTTGGTGTTCCGGGC
GTCGGTGTCCCGGGTAAAGGCGTTCCGGGTGTTGGTGTTCCGGGTGTTGGCGTGGGCGCGGGTG
CCGGCAGCGGTGCAGGCGCTGGTTCTGGCGCTGGCGCGGGTTCCGGTGCCGGTGCAGGCTCCGG
CGCCGGCGCTGGTTCTGGCGTGGGCGCGGGTGCCGGCAGCGGTGCAGGCGCTGGTTCTGGCGCT
GGCGCGGGTTCCGGTGCCGGTGCAGGCTCCGGCGCCGGCGCTGGTTCTGGCGCGAAACCGAGCT
ATCCGCCGACCTATAAAGCCAAACCGTCTTATCCGCCGACGTATAAAGCAAAACCGAGCTATCC
GCCGACGTATAAAGCGAAACCGAGCTATCCGCCGACCTATAAAGCCAAACCGTCTTATCCGCCG
ACGTATAAAGCAAAACCGAGCTATCCGCCGACGTATAAAGCGAAACCGAGCTATCCGCCGACCT
ATAAAGCCAAACCGTCTTATCCGCCGACGTATAAAGCAAAACCGAGCTATCCGCCGACGTATAA
AGCGAAACCGAGCTATCCGCCGACCTATAAAGCCAAACCGTCTTATCCGCCGACGTATAAAGCA
AAACCGAGCTATCCGCCGACGTATAAAGCGAAACCGAGCTATCCGCCGACCTATAAAGCCAAAC
CGTCTTATCCGCCGACGTATAAAGCAAAACCGAGCTATCCGCCGACGTATAAAGTGCCGGGCGT
TGGTGTTCCGGGCGTCGGTGTCCCGGGTAAAGGCGTTCCGGGTGTTGGTGTTCCGGGTGTTGGCG
TGCCGGGCGTTGGTGTTCCGGGCGTCGGTGTCCCGGGTAAAGGCGTTCCGGGTGTTGGTGTTCCG
GGTGTTGGCGTGCCGGGCGTTGGTGTTCCGGGCGTCGGTGTCCCGGGTAAAGGCGTTCCGGGTGT
TGGTGTTCCGGGTGTTGGCGTGCCGGGCGTTGGTGTTCCGGGCGTCGGTGTCCCGGGTAAAGGCG
TTCCGGGTGTTGGTGTTCCGGGTGTTGGCGTGCCGGGCGTTGGTGTTCCGGGCGTCGGTGTCCCG

GGTAAAGGCGTTCCGGGTGTTGGTGTTCCGGGTGTTGGCTATGCGGTGACCGGTCGTGGTGATAG
CCCGGCCTCTAGCGGCGTGCCGGGCGTTGGTGTTCCGGGCGTCGGTGTCCCGGGTAAAGGCGTTC
CGGGTGTTGGTGTTCCGGGTGTTGGCGTGCCGGGCGTTGGTGTTCCGGGCGTCGGTGTCCCGGGT
AAAGGCGTTCCGGGTGTTGGTGTTCCGGGTGTTGGCGTGCCGGGCGTTGGTGTTCCGGGCGTCGG
TGTCCCGGGTAAAGGCGTTCCGGGTGTTGGTGTTCCGGGTGTTGGCGTGCCGGGCGTTGGTGTTC
CGGGCGTCGGTGTCCCGGGTAAAGGCGTTCCGGGTGTTGGTGTTCCGGGTGTTGGCGTGCCGGG
CGTTGGTGTTCCGGGCGTCGGTGTCCCGGGTAAAGGCGTTCCGGGTGTTGGTGTTCCGGGTGTTG
GCGTGGGCGCGGGTGCCGGCAGCGGTGCAGGCGCTGGTTCTGGCGCTGGCGCGGGTTCCGGTGC
CGGTGCAGGCTCCGGCGCCGGCGCTGGTTCTGGCGTGGGCGCGGGTGCCGGCAGCGGTGCAGGC
GCTGGTTCTGGCGCTGGCGCGGGTTCCGGTGCCGGTGCAGGCTCCGGCGCCGGCGCTGGTTCTGG
CGCGAAACCGAGCTATCCGCCGACCTATAAAGCCAAACCGTCTTATCCGCCGACGTATAAAGCA
AAACCGAGCTATCCGCCGACGTATAAAGCGAAACCGAGCTATCCGCCGACCTATAAAGCCAAAC
CGTCTTATCCGCCGACGTATAAAGCAAAACCGAGCTATCCGCCGACGTATAAAGCGAAACCGAG
CTATCCGCCGACCTATAAAGCCAAACCGTCTTATCCGCCGACGTATAAAGCAAAACCGAGCTAT
CCGCCGACGTATAAAGCGAAACCGAGCTATCCGCCGACCTATAAAGCCAAACCGTCTTATCCGC
CGACGTATAAAGCAAAACCGAGCTATCCGCCGACGTATAAAGCGAAACCGAGCTATCCGCCGAC
CTATAAAGCCAAACCGTCTTATCCGCCGACGTATAAAGCAAAACCGAGCTATCCGCCGACGTAT
AAAGCGAAACCGAGCTATCCGCCGACCTATAAAGCGAAACCGAGCTATCCGCCGACCTATAAAG
GCAGCGGTTCTGGTGGCTTCAGCCATCACCATCACCATCACTAATAA

Amino acid sequence (1002 aa):

MVSLLPVRSGVGVPGVGVPGVGVPGKGVPGVGVPGVGVPGVGVPGVGVPGKGVPGVGVPGVGVP
GVGVPGVGVPGKGVPGVGVPGVGVPGVGVPGVGVPGKGVPGVGVPGVGVPGVGVPGVGVPGKGV
PGVGVPGVGYAVTGRGDSPASSGVPGVGVPGVGVPGKGVPGVGVPGVGVPGVGVPGVGVPGKGVP
GVGVPGVGVPGVGVPGVGVPGKGVPGVGVPGVGVPGVGVPGVGVPGKGVPGVGVPGVGVPGVGV
PGVGVPGKGVPGVGVPGVGVGAGAGSGAGAGSGAGAGSGAGAGSGAGAGSGVGAGAGSGAGAG
SGAGAGSGAGAGSGAGAGSGAKPSYPPTYKAKPSYPPTYKAKPSYPPTYKAKPSYPPTYKAKPSYPP
TYKAKPSYPPTYKAKPSYPPTYKAKPSYPPTYKAKPSYPPTYKAKPSYPPTYKAKPSYPPTYKAKPSY
PPTYKAKPSYPPTYKAKPSYPPTYKAKPSYPPTYKVPGVGVPGVGVPGKGVPGVGVPGVGVPGVGV
PGVGVPGKGVPGVGVPGVGVPGVGVPGVGVPGKGVPGVGVPGVGVPGVGVPGVGVPGKGVPGVG
VPGVGVPGVGVPGVGVPGKGVPGVGVPGVGYAVTGRGDSPASSGVPGVGVPGVGVPGKGVPGVG
VPGVGVPGVGVPGVGVPGKGVPGVGVPGVGVPGVGVPGVGVPGKGVPGVGVPGVGVPGVGVPGV
GVPGKGVPGVGVPGVGVPGVGVPGVGVPGKGVPGVGVPGVGVGAGAGSGAGAGSGAGAGSGAG
AGSGAGAGSGVGAGAGSGAGAGSGAGAGSGAGAGSGAGAGSGAKPSYPPTYKAKPSYPPTYKAKP
SYPPTYKAKPSYPPTYKAKPSYPPTYKAKPSYPPTYKAKPSYPPTYKAKPSYPPTYKAKPSYPPTYKA
KPSYPPTYKAKPSYPPTYKAKPSYPPTYKAKPSYPPTYKAKPSYPPTYKAKPSYPPTYKAKPSYPPTY
KAKPSYPPTYKGSGSGGFSHHHHHH

[Laminin peptide-(ELP$_{10}$-Silk$_2$-Mussel$_{15}$)$_2$-Mussel-6xHis]

Nucleotide sequence (2964 bp):

ATGGTTAGCCTGTTGCCGGTGAGGAGCGGTGTGGGCTATCATTACGTGACCATTACGCTGGATTT
ACAGCAAGTGCCGGGCGTTGGTGTTCCGGGCGTCGGTGTCCCGGGTAAAGGCGTTCCGGGTGTT
GGTGTTCCGGGTGTTGGCGTGCCGGGCGTTGGTGTTCCGGGCGTCGGTGTCCCGGGTAAAGGCGT
TCCGGGTGTTGGTGTTCCGGGTGTTGGCGTGCCGGGCGTTGGTGTTCCGGGCGTCGGTGTCCCGG
GTAAAGGCGTTCCGGGTGTTGGTGTTCCGGGTGTTGGCGTGCCGGGCGTTGGTGTTCCGGGCGTC
GGTGTCCCGGGTAAAGGCGTTCCGGGTGTTGGTGTTCCGGGTGTTGGCGTGCCGGGCGTTGGTGT
TCCGGGCGTCGGTGTCCCGGGTAAAGGCGTTCCGGGTGTTGGTGTTCCGGGTGTTGGCGTGCCGG
GCGTTGGTGTTCCGGGCGTCGGTGTCCCGGGTAAAGGCGTTCCGGGTGTTGGTGTTCCGGGTGTT
GGCGTGCCGGGCGTTGGTGTTCCGGGCGTCGGTGTCCCGGGTAAAGGCGTTCCGGGTGTTGGTGT
TCCGGGTGTTGGCGTGCCGGGCGTTGGTGTTCCGGGCGTCGGTGTCCCGGGTAAAGGCGTTCCGG
GTGTTGGTGTTCCGGGTGTTGGCGTGCCGGGCGTTGGTGTTCCGGGCGTCGGTGTCCCGGGTAAA
GGCGTTCCGGGTGTTGGTGTTCCGGGTGTTGGCGTGCCGGGCGTTGGTGTTCCGGGCGTCGGTGT
CCCGGGTAAAGGCGTTCCGGGTGTTGGTGTTCCGGGTGTTGGCGTGGGCGCGGGTGCCGGCAGC
GGTGCAGGCGCTGGTTCTGGCGCTGGCGCGGGTTCCGGTGCCGGTGCAGGCTCCGGCGCCGGCG
CTGGTTCTGGCGTGGGCGCGGGTGCCGGCAGCGGTGCAGGCGCTGGTTCTGGCGCTGGCGCGGG
TTCCGGTGCCGGTGCAGGCTCCGGCGCCGGCGCTGGTTCTGGCGCGAAACCGAGCTATCCGCCG
ACCTATAAAGCCAAACCGTCTTATCCGCCGACGTATAAAGCAAAACCGAGCTATCCGCCGACGT
ATAAAGCGAAACCGAGCTATCCGCCGACCTATAAAGCCAAACCGTCTTATCCGCCGACGTATAA
AGCAAAACCGAGCTATCCGCCGACGTATAAAGCGAAACCGAGCTATCCGCCGACCTATAAAGCC
AAACCGTCTTATCCGCCGACGTATAAAGCAAAACCGAGCTATCCGCCGACGTATAAAGCGAAAC
CGAGCTATCCGCCGACCTATAAAGCCAAACCGTCTTATCCGCCGACGTATAAAGCAAAACCGAG
CTATCCGCCGACGTATAAAGCGAAACCGAGCTATCCGCCGACCTATAAAGCCAAACCGTCTTAT
CCGCCGACGTATAAAGCAAAACCGAGCTATCCGCCGACGTATAAAGTGCCGGGCGTTGGTGTTC
CGGGCGTCGGTGTCCCGGGTAAAGGCGTTCCGGGTGTTGGTGTTCCGGGTGTTGGCGTGCCGGG
CGTTGGTGTTCCGGGCGTCGGTGTCCCGGGTAAAGGCGTTCCGGGTGTTGGTGTTCCGGGTGTTG
GCGTGCCGGGCGTTGGTGTTCCGGGCGTCGGTGTCCCGGGTAAAGGCGTTCCGGGTGTTGGTGTT
CCGGGTGTTGGCGTGCCGGGCGTTGGTGTTCCGGGCGTCGGTGTCCCGGGTAAAGGCGTTCCGG
GTGTTGGTGTTCCGGGTGTTGGCGTGCCGGGCGTTGGTGTTCCGGGCGTCGGTGTCCCGGGTAAA
GGCGTTCCGGGTGTTGGTGTTCCGGGTGTTGGCGTGCCGGGCGTTGGTGTTCCGGGCGTCGGTGT
CCCGGGTAAAGGCGTTCCGGGTGTTGGTGTTCCGGGTGTTGGCGTGCCGGGCGTTGGTGTTCCGG
GCGTCGGTGTCCCGGGTAAAGGCGTTCCGGGTGTTGGTGTTCCGGGTGTTGGCGTGCCGGGCGTT
GGTGTTCCGGGCGTCGGTGTCCCGGGTAAAGGCGTTCCGGGTGTTGGTGTTCCGGGTGTTGGCGT
GCCGGGCGTTGGTGTTCCGGGCGTCGGTGTCCCGGGTAAAGGCGTTCCGGGTGTTGGTGTTCCGG
GTGTTGGCGTGCCGGGCGTTGGTGTTCCGGGCGTCGGTGTCCCGGGTAAAGGCGTTCCGGGTGTT
GGTGTTCCGGGTGTTGGCGTGGGCGCGGGTGCCGGCAGCGGTGCAGGCGCTGGTTCTGGCGCTG
GCGCGGGTTCCGGTGCCGGTGCAGGCTCCGGCGCCGGCGCTGGTTCTGGCGTGGGCGCGGGTGC
CGGCAGCGGTGCAGGCGCTGGTTCTGGCGCTGGCGCGGGTTCCGGTGCCGGTGCAGGCTCCGGC
GCCGGCGCTGGTTCTGGCGCGAAACCGAGCTATCCGCCGACCTATAAAGCCAAACCGTCTTATC
CGCCGACGTATAAAGCAAAACCGAGCTATCCGCCGACGTATAAAGCGAAACCGAGCTATCCGCC
GACCTATAAAGCCAAACCGTCTTATCCGCCGACGTATAAAGCAAAACCGAGCTATCCGCCGACG
TATAAAGCGAAACCGAGCTATCCGCCGACCTATAAAGCCAAACCGTCTTATCCGCCGACGTATA
AAGCAAAACCGAGCTATCCGCCGACGTATAAAGCGAAACCGAGCTATCCGCCGACCTATAAAGC
CAAACCGTCTTATCCGCCGACGTATAAAGCAAAACCGAGCTATCCGCCGACGTATAAAGCGAAA
CCGAGCTATCCGCCGACCTATAAAGCCAAACCGTCTTATCCGCCGACGTATAAAGCAAAACCGA
GCTATCCGCCGACGTATAAAGCGAAACCGAGCTATCCGCCGACCTATAAAGCGAAACCGAGCTA
TCCGCCGACCTATAAAGGCAGCGGTTCTGGTGGCTTCAGCCATCACCATCACCATCACTAATAA

Amino acid sequence (986 aa):

MVSLLPVRSGVGYHYVTITLDLQQVPGVGVPGVGVPGKGVPGVGVPGVGVPGVGVPGVGVPGKGV
PGVGVPGVGVPGVGVPGVGVPGKGVPGVGVPGVGVPGVGVPGVGVPGKGVPGVGVPGVGVPGVG
VPGVGVPGKGVPGVGVPGVGVPGVGVPGVGVPGKGVPGVGVPGVGVPGVGVPGVGVPGKGVPGV
GVPGVGVPGVGVPGVGVPGKGVPGVGVPGVGVPGVGVPGVGVPGKGVPGVGVPGVGVPGVGVPG
VGVPGKGVPGVGVPGVGVGAGAGSGAGAGSGAGAGSGAGAGSGAGAGSGVGAGAGSGAGAGSG
AGAGSGAGAGSGAGAGSGAKPSYPPTYKAKPSYPPTYKAKPSYPPTYKAKPSYPPTYKAKPSYPPTY
KAKPSYPPTYKAKPSYPPTYKAKPSYPPTYKAKPSYPPTYKAKPSYPPTYKAKPSYPPTYKAKPSYPP
TYKAKPSYPPTYKAKPSYPPTYKAKPSYPPTYKVPGVGVPGVGVPGKGVPGVGVPGVGVPGVGVPG
VGVPGKGVPGVGVPGVGVPGVGVPGVGVPGKGVPGVGVPGVGVPGVGVPGVGVPGKGVPGVGVP
GVGVPGVGVPGVGVPGKGVPGVGVPGVGVPGVGVPGVGVPGKGVPGVGVPGVGVPGVGVPGVGV
PGKGVPGVGVPGVGVPGVGVPGVGVPGKGVPGVGVPGVGVPGVGVPGVGVPGKGVPGVGVPGVG
VPGVGVPGVGVPGKGVPGVGVPGVGVGAGAGSGAGAGSGAGAGSGAGAGSGAGAGSGVGAGAG
SGAGAGSGAGAGSGAGAGSGAGAGSGAKPSYPPTYKAKPSYPPTYKAKPSYPPTYKAKPSYPPTYK
AKPSYPPTYKAKPSYPPTYKAKPSYPPTYKAKPSYPPTYKAKPSYPPTYKAKPSYPPTYKAKPSYPPT
YKAKPSYPPTYKAKPSYPPTYKAKPSYPPTYKAKPSYPPTYKAKPSYPPTYKAKPSYPPTYKGSGSGG
FSHHHHHH

[Heparin-binding peptide-(ELP_{10}-$Silk_2$-$Mussel_{15}$)$_2$-Mussel-6xHis]

Nucleotide sequence (2973 bp):

ATGGTTAGCCTGTTGCCGGTGAGGAGCGGTGTGGGCTATCCGACCCAGCGTGCGCGCTATCAAT
GGGTGCGTTGCAACCCGGTGCCGGGCGTTGGTGTTCCGGGCGTCGGTGTCCCGGGTAAAGGCGT
TCCGGGTGTTGGTGTTCCGGGTGTTGGCGTGCCGGGCGTTGGTGTTCCGGGCGTCGGTGTCCCGG
GTAAAGGCGTTCCGGGTGTTGGTGTTCCGGGTGTTGGCGTGCCGGGCGTTGGTGTTCCGGGCGTC
GGTGTCCCGGGTAAAGGCGTTCCGGGTGTTGGTGTTCCGGGTGTTGGCGTGCCGGGCGTTGGTGT
TCCGGGCGTCGGTGTCCCGGGTAAAGGCGTTCCGGGTGTTGGTGTTCCGGGTGTTGGCGTGCCGG
GCGTTGGTGTTCCGGGCGTCGGTGTCCCGGGTAAAGGCGTTCCGGGTGTTGGTGTTCCGGGTGTT
GGCGTGCCGGGCGTTGGTGTTCCGGGCGTCGGTGTCCCGGGTAAAGGCGTTCCGGGTGTTGGTGT
TCCGGGTGTTGGCGTGCCGGGCGTTGGTGTTCCGGGCGTCGGTGTCCCGGGTAAAGGCGTTCCGG
GTGTTGGTGTTCCGGGTGTTGGCGTGCCGGGCGTTGGTGTTCCGGGCGTCGGTGTCCCGGGTAAA
GGCGTTCCGGGTGTTGGTGTTCCGGGTGTTGGCGTGCCGGGCGTTGGTGTTCCGGGCGTCGGTGT
CCCGGGTAAAGGCGTTCCGGGTGTTGGTGTTCCGGGTGTTGGCGTGCCGGGCGTTGGTGTTCCGG
GCGTCGGTGTCCCGGGTAAAGGCGTTCCGGGTGTTGGTGTTCCGGGTGTTGGCGTGGGCGCGGG
TGCCGGCAGCGGTGCAGGCGCTGGTTCTGGCGCTGGCGCGGGTTCCGGTGCCGGTGCAGGCTCC
GGCGCCGGCGCTGGTTCTGGCGTGGGCGCGGGTGCCGGCAGCGGTGCAGGCGCTGGTTCTGGCG
CTGGCGCGGGTTCCGGTGCCGGTGCAGGCTCCGGCGCCGGCGCTGGTTCTGGCGCGAAACCGAG
CTATCCGCCGACCTATAAAGCCAAACCGTCTTATCCGCCGACGTATAAAGCAAAACCGAGCTAT
CCGCCGACGTATAAAGCGAAACCGAGCTATCCGCCGACCTATAAAGCCAAACCGTCTTATCCGC
CGACGTATAAAGCAAAACCGAGCTATCCGCCGACGTATAAAGCGAAACCGAGCTATCCGCCGAC
CTATAAAGCCAAACCGTCTTATCCGCCGACGTATAAAGCAAAACCGAGCTATCCGCCGACGTAT
AAAGCGAAACCGAGCTATCCGCCGACCTATAAAGCCAAACCGTCTTATCCGCCGACGTATAAAG
CAAAACCGAGCTATCCGCCGACGTATAAAGCGAAACCGAGCTATCCGCCGACCTATAAAGCCAA
ACCGTCTTATCCGCCGACGTATAAAGCAAAACCGAGCTATCCGCCGACGTATAAAGTGCCGGGC
GTTGGTGTTCCGGGCGTCGGTGTCCCGGGTAAAGGCGTTCCGGGTGTTGGTGTTCCGGGTGTTGG
CGTGCCGGGCGTTGGTGTTCCGGGCGTCGGTGTCCCGGGTAAAGGCGTTCCGGGTGTTGGTGTTC
CGGGTGTTGGCGTGCCGGGCGTTGGTGTTCCGGGCGTCGGTGTCCCGGGTAAAGGCGTTCCGGG
TGTTGGTGTTCCGGGTGTTGGCGTGCCGGGCGTTGGTGTTCCGGGCGTCGGTGTCCCGGGTAAAG
GCGTTCCGGGTGTTGGTGTTCCGGGTGTTGGCGTGCCGGGCGTTGGTGTTCCGGGCGTCGGTGTC

CCGGGTAAAGGCGTTCCGGGTGTTGGTGTTCCGGGTGTTGGCGTGCCGGGCGTTGGTGTTCCGGG
CGTCGGTGTCCCGGGTAAAGGCGTTCCGGGTGTTGGTGTTCCGGGTGTTGGCGTGCCGGGCGTTG
GTGTTCCGGGCGTCGGTGTCCCGGGTAAAGGCGTTCCGGGTGTTGGTGTTCCGGGTGTTGGCGTG
CCGGGCGTTGGTGTTCCGGGCGTCGGTGTCCCGGGTAAAGGCGTTCCGGGTGTTGGTGTTCCGGG
TGTTGGCGTGCCGGGCGTTGGTGTTCCGGGCGTCGGTGTCCCGGGTAAAGGCGTTCCGGGTGTTG
GTGTTCCGGGTGTTGGCGTGCCGGGCGTTGGTGTTCCGGGCGTCGGTGTCCCGGGTAAAGGCGTT
CCGGGTGTTGGTGTTCCGGGTGTTGGCGTGGGCGCGGGTGCCGGCAGCGGTGCAGGCGCTGGTT
CTGGCGCTGGCGCGGGTTCCGGTGCCGGTGCAGGCTCCGGCGCCGGCGCTGGTTCTGGCGTGGG
CGCGGGTGCCGGCAGCGGTGCAGGCGCTGGTTCTGGCGCTGGCGCGGGTTCCGGTGCCGGTGCA
GGCTCCGGCGCCGGCGCTGGTTCTGGCGCGAAACCGAGCTATCCGCCGACCTATAAAGCCAAAC
CGTCTTATCCGCCGACGTATAAAGCAAAACCGAGCTATCCGCCGACGTATAAAGCGAAACCGAG
CTATCCGCCGACCTATAAAGCCAAACCGTCTTATCCGCCGACGTATAAAGCAAAACCGAGCTAT
CCGCCGACGTATAAAGCGAAACCGAGCTATCCGCCGACCTATAAAGCCAAACCGTCTTATCCGC
CGACGTATAAAGCAAAACCGAGCTATCCGCCGACGTATAAAGCGAAACCGAGCTATCCGCCGAC
CTATAAAGCCAAACCGTCTTATCCGCCGACGTATAAAGCAAAACCGAGCTATCCGCCGACGTAT
AAAGCGAAACCGAGCTATCCGCCGACCTATAAAGCCAAACCGTCTTATCCGCCGACGTATAAAG
CAAAACCGAGCTATCCGCCGACGTATAAAGCGAAACCGAGCTATCCGCCGACCTATAAAGCGAA
ACCGAGCTATCCGCCGACCTATAAAGGCAGCGGTTCTGGTGGCTTCAGCCATCACCATCACCATC
ACTAATAA

Amino acid sequence (989 aa):

MVSLLPVRSGVGYPTQRARYQWVRCNPVPGVGVPGVGVPGKGVPGVGVPGVGVPGVGVPGVGVP
GKGVPGVGVPGVGVPGVGVPGVGVPGKGVPGVGVPGVGVPGVGVPGVGVPGKGVPGVGVPGVGV
PGVGVPGVGVPGKGVPGVGVPGVGVPGVGVPGVGVPGKGVPGVGVPGVGVPGVGVPGVGVPGKG
VPGVGVPGVGVPGVGVPGVGVPGKGVPGVGVPGVGVPGVGVPGVGVPGKGVPGVGVPGVGVPGV
GVPGVGVPGKGVPGVGVPGVGVGAGAGSGAGAGSGAGAGSGAGAGSGAGAGSGVGAGAGSGAG
AGSGAGAGSGAGAGSGAGAGSGAKPSYPPTYKAKPSYPPTYKAKPSYPPTYKAKPSYPPTYKAKPSY
PPTYKAKPSYPPTYKAKPSYPPTYKAKPSYPPTYKAKPSYPPTYKAKPSYPPTYKAKPSYPPTYKAKP
SYPPTYKAKPSYPPTYKAKPSYPPTYKAKPSYPPTYKVPGVGVPGVGVPGKGVPGVGVPGVGVPGV
GVPGVGVPGKGVPGVGVPGVGVPGVGVPGVGVPGKGVPGVGVPGVGVPGVGVPGVGVPGKGVPG
VGVPGVGVPGVGVPGVGVPGKGVPGVGVPGVGVPGVGVPGVGVPGKGVPGVGVPGVGVPGVGVP
GVGVPGKGVPGVGVPGVGVPGVGVPGVGVPGKGVPGVGVPGVGVPGVGVPGVGVPGKGVPGVGV
PGVGVPGVGVPGVGVPGKGVPGVGVPGVGVGAGAGSGAGAGSGAGAGSGAGAGSGAGAGSGVG
AGAGSGAGAGSGAGAGSGAGAGSGAGAGSGAKPSYPPTYKAKPSYPPTYKAKPSYPPTYKAKPSYP
PTYKAKPSYPPTYKAKPSYPPTYKAKPSYPPTYKAKPSYPPTYKAKPSYPPTYKAKPSYPPTYKAKPS
YPPTYKAKPSYPPTYKAKPSYPPTYKAKPSYPPTYKAKPSYPPTYKAKPSYPPTYKAKPSYPPTYKGS
GSGGFSHHHHHH

[BMP-2 peptide-(ELP$_{10}$-Silk$_2$-Mussel$_{15}$)$_2$-Mussel-6xHis]

Nucleotide sequence (2958 bp):

ATGGTTAGCCTGTTGCCGGTGAGGAGCGGTGTGGGCGCGATTAGCATGCTGTATTTAGATGAAA
ACGTGCCGGGCGTTGGTGTTCCGGGCGTCGGTGTCCCGGGTAAAGGCGTTCCGGGTGTTGGTGTT
CCGGGTGTTGGCGTGCCGGGCGTTGGTGTTCCGGGCGTCGGTGTCCCGGGTAAAGGCGTTCCGG
GTGTTGGTGTTCCGGGTGTTGGCGTGCCGGGCGTTGGTGTTCCGGGCGTCGGTGTCCCGGGTAAA
GGCGTTCCGGGTGTTGGTGTTCCGGGTGTTGGCGTGCCGGGCGTTGGTGTTCCGGGCGTCGGTGT

CCCGGGTAAAGGCGTTCCGGGTGTTGGTGTTCCGGGTGTTGGCGTGCCGGGCGTTGGTGTTCCGG
GCGTCGGTGTCCCGGGTAAAGGCGTTCCGGGTGTTGGTGTTCCGGGTGTTGGCGTGCCGGGCGTT
GGTGTTCCGGGCGTCGGTGTCCCGGGTAAAGGCGTTCCGGGTGTTGGTGTTCCGGGTGTTGGCGT
GCCGGGCGTTGGTGTTCCGGGCGTCGGTGTCCCGGGTAAAGGCGTTCCGGGTGTTGGTGTTCCGG
GTGTTGGCGTGCCGGGCGTTGGTGTTCCGGGCGTCGGTGTCCCGGGTAAAGGCGTTCCGGGTGTT
GGTGTTCCGGGTGTTGGCGTGCCGGGCGTTGGTGTTCCGGGCGTCGGTGTCCCGGGTAAAGGCGT
TCCGGGTGTTGGTGTTCCGGGTGTTGGCGTGCCGGGCGTTGGTGTTCCGGGCGTCGGTGTCCCGG
GTAAAGGCGTTCCGGGTGTTGGTGTTCCGGGTGTTGGCGTGGGCGCGGGTGCCGGCAGCGGTGC
AGGCGCTGGTTCTGGCGCTGGCGCGGGTTCCGGTGCCGGTGCAGGCTCCGGCGCCGGCGCTGGT
TCTGGCGTGGGCGCGGGTGCCGGCAGCGGTGCAGGCGCTGGTTCTGGCGCTGGCGCGGGTTCCG
GTGCCGGTGCAGGCTCCGGCGCCGGCGCTGGTTCTGGCGCGAAACCGAGCTATCCGCCGACCTA
TAAAGCCAAACCGTCTTATCCGCCGACGTATAAAGCAAAACCGAGCTATCCGCCGACGTATAAA
GCGAAACCGAGCTATCCGCCGACCTATAAAGCCAAACCGTCTTATCCGCCGACGTATAAAGCAA
AACCGAGCTATCCGCCGACGTATAAAGCGAAACCGAGCTATCCGCCGACCTATAAAGCCAAACC
GTCTTATCCGCCGACGTATAAAGCAAAACCGAGCTATCCGCCGACGTATAAAGCGAAACCGAGC
TATCCGCCGACCTATAAAGCCAAACCGTCTTATCCGCCGACGTATAAAGCAAAACCGAGCTATC
CGCCGACGTATAAAGCGAAACCGAGCTATCCGCCGACCTATAAAGCCAAACCGTCTTATCCGCC
GACGTATAAAGCAAAACCGAGCTATCCGCCGACGTATAAAGTGCCGGGCGTTGGTGTTCCGGGC
GTCGGTGTCCCGGGTAAAGGCGTTCCGGGTGTTGGTGTTCCGGGTGTTGGCGTGCCGGGCGTTGG
TGTTCCGGGCGTCGGTGTCCCGGGTAAAGGCGTTCCGGGTGTTGGTGTTCCGGGTGTTGGCGTGC
CGGGCGTTGGTGTTCCGGGCGTCGGTGTCCCGGGTAAAGGCGTTCCGGGTGTTGGTGTTCCGGGT
GTTGGCGTGCCGGGCGTTGGTGTTCCGGGCGTCGGTGTCCCGGGTAAAGGCGTTCCGGGTGTTGG
TGTTCCGGGTGTTGGCGTGCCGGGCGTTGGTGTTCCGGGCGTCGGTGTCCCGGGTAAAGGCGTTC
CGGGTGTTGGTGTTCCGGGTGTTGGCGTGCCGGGCGTTGGTGTTCCGGGCGTCGGTGTCCCGGGT
AAAGGCGTTCCGGGTGTTGGTGTTCCGGGTGTTGGCGTGCCGGGCGTTGGTGTTCCGGGCGTCGG
TGTCCCGGGTAAAGGCGTTCCGGGTGTTGGTGTTCCGGGTGTTGGCGTGCCGGGCGTTGGTGTTC
CGGGCGTCGGTGTCCCGGGTAAAGGCGTTCCGGGTGTTGGTGTTCCGGGTGTTGGCGTGCCGGG
CGTTGGTGTTCCGGGCGTCGGTGTCCCGGGTAAAGGCGTTCCGGGTGTTGGTGTTCCGGGTGTTG
GCGTGCCGGGCGTTGGTGTTCCGGGCGTCGGTGTCCCGGGTAAAGGCGTTCCGGGTGTTGGTGTT
CCGGGTGTTGGCGTGGGCGCGGGTGCCGGCAGCGGTGCAGGCGCTGGTTCTGGCGCTGGCGCGG
GTTCCGGTGCCGGTGCAGGCTCCGGCGCCGGCGCTGGTTCTGGCGTGGGCGCGGGTGCCGGCAG
CGGTGCAGGCGCTGGTTCTGGCGCTGGCGCGGGTTCCGGTGCCGGTGCAGGCTCCGGCGCCGGC
GCTGGTTCTGGCGCGAAACCGAGCTATCCGCCGACCTATAAAGCCAAACCGTCTTATCCGCCGA
CGTATAAAGCAAAACCGAGCTATCCGCCGACGTATAAAGCGAAACCGAGCTATCCGCCGACCTA
TAAAGCCAAACCGTCTTATCCGCCGACGTATAAAGCAAAACCGAGCTATCCGCCGACGTATAAA
GCGAAACCGAGCTATCCGCCGACCTATAAAGCCAAACCGTCTTATCCGCCGACGTATAAAGCAA
AACCGAGCTATCCGCCGACGTATAAAGCGAAACCGAGCTATCCGCCGACCTATAAAGCCAAACC
GTCTTATCCGCCGACGTATAAAGCAAAACCGAGCTATCCGCCGACGTATAAAGCGAAACCGAGC
TATCCGCCGACCTATAAAGCCAAACCGTCTTATCCGCCGACGTATAAAGCAAAACCGAGCTATC
CGCCGACGTATAAAGCGAAACCGAGCTATCCGCCGACCTATAAAGCGAAACCGAGCTATCCGCC
GACCTATAAAGGCAGCGGTTCTGGTGGCTTCAGCCATCACCATCACCATCACTAATAA

Amino acid sequence (984 aa):

MVSLLPVRSGVGAISMLYLDENVPGVGVPGVGVPGKGVPGVGVPGVGVPGVGVPGVGVPGKGVPG
VGVPGVGVPGVGVPGVGVPGKGVPGVGVPGVGVPGVGVPGVGVPGKGVPGVGVPGVGVPGVGVP
GVGVPGKGVPGVGVPGVGVPGVGVPGVGVPGKGVPGVGVPGVGVPGVGVPGVGVPGKGVPGVGV
PGVGVPGVGVPGVGVPGKGVPGVGVPGVGVPGVGVPGVGVPGKGVPGVGVPGVGVPGVGVPGVG
VPGKGVPGVGVPGVGVGAGAGSGAGAGSGAGAGSGAGAGSGAGAGSGVGAGAGSGAGAGSGAG
AGSGAGAGSGAGAGSGAKPSYPPTYKAKPSYPPTYKAKPSYPPTYKAKPSYPPTYKAKPSYPPTYKA

KPSYPPTYKAKPSYPPTYKAKPSYPPTYKAKPSYPPTYKAKPSYPPTYKAKPSYPPTYKAKPSYPPTY
KAKPSYPPTYKAKPSYPPTYKAKPSYPPTYKVPGVGVPGVGVPGKGVPGVGVPGVGVPGVGVPGVG
VPGKGVPGVGVPGVGVPGVGVPGVGVPGKGVPGVGVPGVGVPGVGVPGVGVPGKGVPGVGVPGV
GVPGVGVPGVGVPGKGVPGVGVPGVGVPGVGVPGVGVPGKGVPGVGVPGVGVPGVGVPGVGVPG
KGVPGVGVPGVGVPGVGVPGVGVPGKGVPGVGVPGVGVPGVGVPGVGVPGKGVPGVGVPGVGVP
GVGVPGVGVPGKGVPGVGVPGVGVGAGAGSGAGAGSGAGAGSGAGAGSGAGAGSGVGAGAGSG
AGAGSGAGAGSGAGAGSGAGAGSGAKPSYPPTYKAKPSYPPTYKAKPSYPPTYKAKPSYPPTYKAK
PSYPPTYKAKPSYPPTYKAKPSYPPTYKAKPSYPPTYKAKPSYPPTYKAKPSYPPTYKAKPSYPPTYK
AKPSYPPTYKAKPSYPPTYKAKPSYPPTYKAKPSYPPTYKAKPSYPPTYKAKPSYPPTYKGSGSGGFS
HHHHHH
